# Identification and Functional Divergence Analysis of *WOX* Gene Family in Paper Mulberry

**DOI:** 10.3390/ijms18081782

**Published:** 2017-08-16

**Authors:** Feng Tang, Naizhi Chen, Meiling Zhao, Yucheng Wang, Ruiping He, Xianjun Peng, Shihua Shen

**Affiliations:** 1 Key Laboratory of Plant Resources, Institute of Botany, The Chinese Academy of Sciences, Beijing 100093, China; tangfeng76@ibcas.ac.cn (F.T.); chennaizhi@ibcas.ac.cn (N.C.); zml@ibcas.ac.cn (M.Z.); wangyucheng731@berrygenomics.com (Y.W.); heruipinging@126.com (R.H.); 2 University of Chinese Academy of Sciences, Beijing 100049, China

**Keywords:** WOX transcription factor, paper mulberry, development, phytohormone, stress response

## Abstract

The WOX (WUSCHEL-related homeobox) is a plant-specific transcription factor involved in plant development and stress response. However, few studies have been reported on the *WOX* gene in woody plants. In this study, 10 *BpWOX* genes were isolated from paper mulberry by RACE-PCR and categorized into three clades through phylogenetic analysis, ancient, intermediate and WUS clade. Among them, five members had the transcriptional activity detected by yeast one-hybrid and seven were uniquely localized to the nucleus through green fluorescent protein (GFP) observation. The expression patterns of *BpWOX* genes in different tissues and under diverse treatments were quantified by the qRT-PCR method. Results showed that *BpWUS* was expressed in the apical bud, stem and root, *BpWOX5* and *BpWOX7* functioned only in the root tip, and three *BpWOXs* regulated leaf development redundantly. *BpWOX9* and *BpWOX10* were induced by indole-3-acetic acid (IAA) or jasmonic acid (JA), while *BpWOX2* was repressed by five phytohormones. Interestingly, most *BpWOX* genes were responsive to the abiotic stress stimuli of drought, salt, cold, and cadmium (CdCl_2_). Together, our study revealed that BpWOXs were functionally divergent during paper mulberry development and environmental adaptation, which might be related to their evolutionary relationships. Our work will benefit the systematic understanding of the precise function of WOX in plant development and environmental stress responses.

## 1. Introduction

The WOX protein is from the homeobox (HOX) super family [1], and consists of 65 amino acids folding into a DNA-binding domain with three helixes in space [2]. The homeodomain of the HOX family is about 60 amino acids; the WOX family has one or two extra residues between helices 1 and 2, and four to five extra residues between helices 2 and 3 [1]. The *WOX* gene is a plant-specific transcription factor; however, study on the transcription activity and nuclear localization of the WOX protein is rare. There has only been one study on the transactivation activity of WOX, where the OsWOX3 had the ability to activate the downstream gene expression [3], and the AtWOX3 [4], AtWOX4 [5], and AtWUS [2] have shown an obvious nuclear localization. Additionally, the AtWOX1 localized in both the nucleus and cytoplasm, suggesting that AtWOX1 not only serves as a transcription factor working in the nucleus, but also possibly implements its function in cytoplasm [6].

The phylogenetic tree of the plant WOX proteins can be categorized into three clades, i.e., the WUS, intermediate and ancient clade [7]. The ancient clade contains the most conserved plant WOX proteins. Except for AtWOX7, the WUS-box is conserved in the modern clade WOX in *Arabidopsis thaliana* [8]. All major radiations within the *WOX* gene family take place before the angiosperm-gymnosperm split and there has been a recent expansion within the intermediate clade in the Pinaceae family [9]. The analysis of *WOX* gene expression and function shows that the WOX family fulfills specialized functions in the developmental processes of plants, such as embryonic patterning, stem-cell maintenance, and lateral organ development. These functions may be related to either the promotion of cell division activity and/or the prevention of premature cell differentiation [7].

In the ancient clade, *AtWOX13* is dynamically expressed during primary and lateral root initiation and development [10]. *AtWOX14* is expressed predominantly in vascular tissue and functions redundantly with *AtWOX4* in the regulation of vascular cell division [11]. *AtWOX14* promotes procambial cell proliferation and differentiation by promoting the accumulation of bioactive gibberellin (GA) [12]. *AtWOX10* may be a pseudogene, as no transcripts have ever been detected [10]. As for the intermediate clade, *AtWOX8* and *AtWOX9* are initially co-expressed in the egg cell and zygote and then confined to the apical and basal daughter cells of the zygote by its asymmetric division, respectively, and are both involved in the early patterning of Arabidopsis embryo development [13]. *OsDWT1*, the homologous of *AtWOX8* and *AtWOX9*, promotes the internode growth in rice by affecting cell division and cell elongation [14]. *AtWOX11*, acting redundantly with its homolog *AtWOX12*, is involved in the first-step cell fate transition during de novo root of organogenesis [15]. In rice, *OsWOX11* is expressed in emerging crown roots, and participates in the activation of crown root emergence and growth [16]. In the WUS clade, the *WUS* gene is the founding member of the *WOX* gene family. *AtWUS* is expressed specifically in the organizing center of the SAM, and it has been proven to play a key role in the stem cell maintenance of shoot apical meristem [17]. *AtWOX5*, a homologue of the *WUS* gene, is expressed in the quiescent center (QC) cells of the root stem cell niche, establishing quiescence by suppressing *CYCLIN D* Activity [18]. *AtWOX5* and *AtWUS* have been shown to be exchangeable in regulating stem-cell maintenance in shoot and root [19]. *AtWOX4* is expressed preferentially in the procambium and cambium, and is required for promoting the proliferation of procambial/cambial stem cells [5,20,21]. *PFS2/AtWOX6*, which is expressed most abundantly in developing ovules, affects either ovule patterning or differentiation [22]. In leaf primordia, *PRESSED FLOWER (PRS)/AtWOX3* and *AtWOX1* are expressed at the adaxial-abaxial boundary layer both in the middle mesophyll and at the leaf margin, suggesting their function in leaf blade outgrowth and margin development [23,24]. The auxin synthesis (*YUC*) and auxin transport (*PIN*) related genes are altered in expression level in the *NAL2/3* double mutant [3], and over expression of OsWOX3A protein (encoded by *NAL2/3*) consistently exhibits severe dwarfism with very short and wide leaves, and this can be rescued by exogenous GA [25]. Ectopic expression of *STENOFOLIA (STF)* in three monocot species, i.e., switchgrass, *Brachypodium distachyon* and rice improves the biomass yield, and STF directly binds to several regions in the promoters of cytokinin oxidase/dehydrogenase genes and represses their transcription allowing accumulation of active cytokinin pools, leading to more leaf blade lateral outgrowth [26]. *OsWOX3A* is reported to participate in root development by modulating GA-auxin crosstalk [27]. These results suggest the regulation of WOX transcription factor in leaf expansion is involved with auxin, cytokinin and gibberellin.

Most *WOX* genes have been proven to be mainly involved in the plant development processes, however, their roles in response to abiotic stress unknown. An additional role for *AtWOX6* in response to cold stress was identified by the isolation of a mutant allele of *AtWOX6* named *HOS9-1*. The mutant grows more slowly, flowers later, and is more sensitive to freezing [28]. In rice, most *WOX* genes are found to be responsive to abiotic stress stimuli of drought, salt or cold [29]. Three *BpWOX* genes were induced by cold treatment after 6 h from the transcriptome sequencing of paper mulberry [30], implying that they also have important roles in cold stress.

Paper mulberry (*Broussonetia kazinoki × Broussonetia papyrifera*), a perennial woody species, belongs to the Moraceae. The paper mulberry is branchy and its leaves enriched with crude proteins, which makes it an ideal woody forage. The bark of the paper mulberry is a good source of fiber for paper making [31]. Due to its rapid growth and strong adaptability, the paper mulberry has been widely used in the ecological afforestation and landscape recovery in mined areas [32]. Its main strategies for undergoing the tough environment are to develop lateral root and modulate leaf morphogenesis. As the WOX proteins play a very important role in the morphogenesis establishment of plants, our study of *BpWOX* genes would provide implication in understanding its role in paper mulberry’s growth and development and its tolerance in different adverse environments.

## 2. Results

### 2.1. Subsection Identification and Structure Analysis of BpWOX Genes

The genomic sequence of paper mulberry has not been published until now. To obtain some fragment sequences of the *BpWOX* gene from the paper mulberry, we designed the degenerate primers to target the conserved region of each *WOX* gene. The sequences of 83 *WOX* genes from 7 species (*Morus notabilis*, *Fragaria vesca*, *Prunus persica*, *Malus domestica*, *Medicago truncatula*, *A. thaliana* and *Oryza sativa*) were aligned to find the conserved region (Supplementary Figures S1–S11). The isolated fragment sequences were further extended by using the rapid amplification of cDNA ends polymerase chain reaction (RACE-PCR) to acquire the full-length cDNA sequences (Supplementary Tables S1–S4). In this study, we have successfully isolated 10 *BpWOX* genes except for *BpWOX6* and the 5′-UTR of *BpWOX7*. For *BpWOX6*, we did not obtain any sequence information from the paper mulberry. The full length of cDNA sequences were submitted to NCBI, and the GenBank accession numbers are displayed in Table 1.

**Figure 1:**
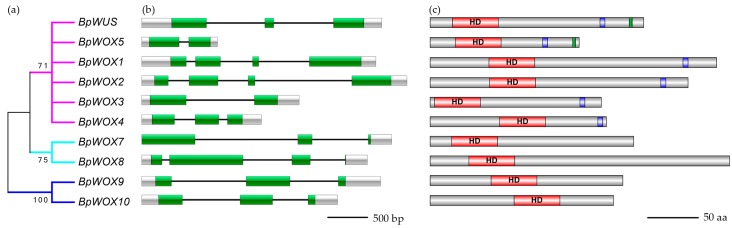
The schematic of *BpWOX* genes structure and homeodomain of BpWOX proteins. (**a**) The phylogenetic tree of *BpWOX* genes was reconstructed based on the multiple sequence alignment by using the neighbor-joining method in MEGA 6.0 with 1000 bootstrap replicates; (**b**) The structures of each *BpWOX* genes were investigated by the alignment of cDNA sequences and corresponding gDNA sequences. The untranslated regions (UTR) are colored with the gray-box, the exons colored with the green-box, and the black lines represent the introns. Bar = 500 bp; (**c**) The length of the amino acid of each BpWOX proteins was colored in gray, the homeodomain (red boxes) was across all proteins, the WUS-box (blue boxes) was only presented in the WUS clade, and the ERA domain (green boxes) were found in BpWUS and BpWOX5. Bar = 50 aa.

**Figure 2:**
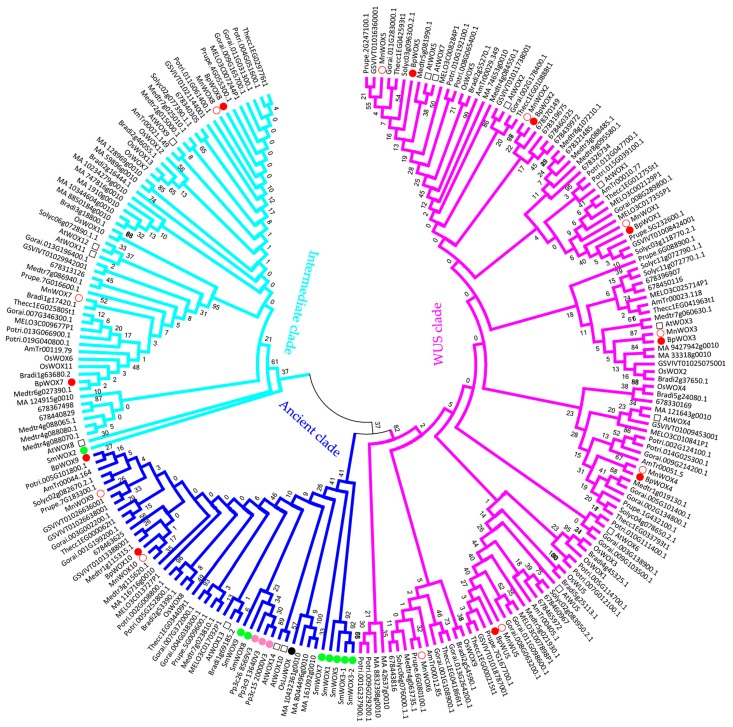
Phylogenetic analysis of plant WOX family. Evolutionary analysis was conducted in MEGA 6.0, and the bootstrap replicates was 1000. A simplified version of the neighbor joining (NJ) tree was displayed, with 228 sequences of proteins from 19 species from green algae to angiosperms. The tree was divided into three clades; the WUS clade, containing 122 sequences, 16 species (pink); the intermediate clade, containing 59 sequences, 17 species (sky-blue); and the ancient clade, containing 47 sequences, 19 species (navy-blue). Two species, *O. lucimarinus* (black filled circle) and *P. patens* (pink filled circle) had members only in the ancient clade. *S. moellendorffii* (green filled circle) had members in both the ancient clade and intermediate clade. Other species had members in all three clades. The paper mulberry (red filled circle) had two members in the ancient clade and intermediate clade, respectively, and six members in the WUS clade. Mulberry is marked with red blank circle, and the *A. thaliana* is marked by the black blank box. The information of species used in the phylogenetic tree is shown in Supplementary Table S6.

The length of genomic DNA of 10 *BpWOX* genes varied from 1 to 3 kb, and the open reading frames were from 600 to 1300 bp (Table 1). The structure of each *BpWOX* genes was investigated by the alignment of cDNA sequences and corresponding gDNA sequences. The results showed that the *BpWOX* genes had at least one intron and four exons maximum, and the last exon of the *BpWOX8* gene was about 6 bp length coding one amino acid and a stop codon (Figure 1b). The phylogenetic analysis of *BpWOX* genes showed that they were categorized into three clades (Figure 1a). The BpWOX9 and BpWOX10 belonged to the ancient clade, BpWOX7 and BpWOX8 belonged to the intermediate clade, and the rest of the BpWOXs belonged to the WUS clade (Figure 2). The amino acid number, the molecular weight, and the isoelectric point of each BpWOX proteins are displayed in Table 1. Each of the BpWOX proteins contained a highly conserved homeodomain, whose position varied across different members of the BpWOX family (Figure 1c); furthermore, the downstream region of the homeodomain in the WUS clade proteins contained a WUS-box motif (T-L-X-L-F-P-X-X). In addition, an ERF-associated amphiphilic repression (EAR) domain was present at the carboxy-terminal ends of BpWUS and BpWOX5 (Figure 1c).

### 2.2. The Phylogenetic Analysis of BpWOX Family

To obtain a better understanding of the evolutionary history and phylogenetic relationships of WOX proteins in paper mulberry, an unrooted phylogenetic tree was constructed with neighbor-joining method on the basis of the multiple sequence alignment of 228 protein sequences from 19 species (Figure 2). From green algae to flowering plants, WOX members were divided into three clades, and each species had at least one WOX member in the ancient clade (Supplementary Table S5). The WUS clade contained members from only seeds plants; the intermediate clade contained members only from vascular plants; and the ancient clade contained members from lower plants to higher plants (Supplementary Table S5). It was interesting that the green algae *Ostreococcus lucimarinus* and non-vascular moss *Physcomitrella patens* were only present in the ancient clade (Figure 2), suggesting that the WOX protein had a monophyletic origin in green algae. The oldest vascular lycophyte *Selaginella moellendorffii* had eight WOX proteins, only SmWOX2 was classified into the intermediate clade, the rest of the SmWOX proteins belonged to the ancient clade (Figure 2). This indicated that the evolution of WOX in the intermediate clade may be involved in vascular development. Compared with the ancient clade, the members of the intermediate clade may have specific functions exclusive to plants. The number of WOX proteins in the WUS clade expanded mainly in gymnosperm and angiosperm plants, suggesting that the WUS clade was specific for the seed plants.

**Figure 3:**
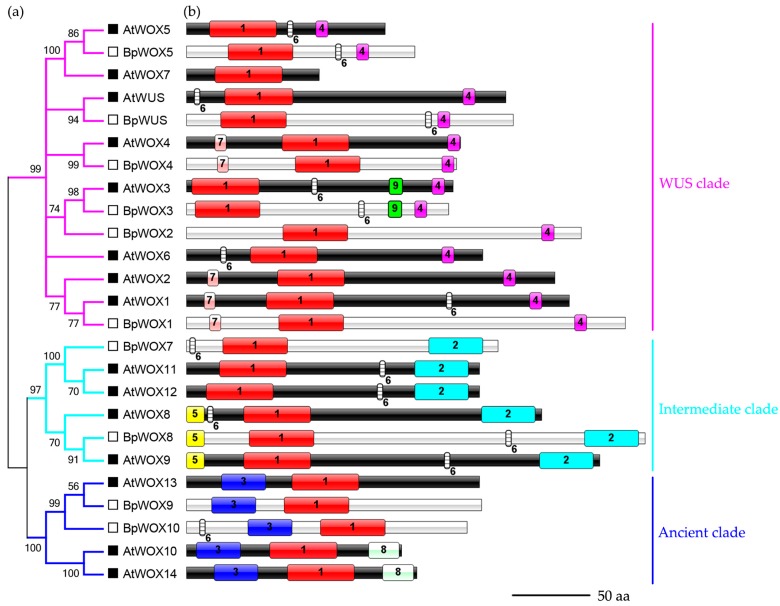
Phylogenetic tree and conserved motif analysis of WOX family between *A. thaliana* and paper mulberry. (**a**) The phylogenetic tree (NJ) was reconstructed by MAGE6 using 24 sequences from *A. thaliana* and paper mulberry. The WUS clade, intermediate clade, and ancient clade were colored with pink, sky-blue and navy blue, respectively. The BpWOX proteins were represented by black filled boxes, and the AtWOX proteins were represented by black blank boxes; (**b**) The conserved motifs among the members are highlighted in colored boxes with an arranged number, and the sequences of the motifs are listed in Supplementary Table S7. A total of nine motifs was observed from the motif elicitation tool MEME (Supplementary Figure S12). Motif 1 (red boxes) was homeodomain (HD) and motif 4 (pink boxes) was the WUS-box. Motif 2 (sky-blue boxes) only appeared in the intermediate clade and motif 3 (navy-blue boxes) was unique to the ancient clade. The other motifs are distinguished by colors except for motif 6 (white plaid). Motif 5 is labeled by yellow boxes, motif 7 is labeled by water-red boxes, motif 8 is labeled by white boxes, and motif 9 is labeled by green boxes. Bar = 50 aa.

To further understand the function of BpWOX proteins, we reconstructed the phylogenetic relationships and compared the conserved motif from 24 protein sequences between *A. thaliana* and *B. papyrifera* (Figure 3). Consistently, these WOX members were also divided into three clades, and each counterpart of BpWOX can be found in *A. thaliana*. The ancient clade contained BpWOX9, BpWOX10, AtWOX13, AtWOX10 and AtWOX14, and AtWOX13 was the orthologous protein to BpWOX9 and BpWOX10. The intermediate clade included two BpWOXs and four AtWOXs, and each BpWOX had two AtWOX orthologs. All members of the WUS clade in *A. thaliana*, except AtWOX2 and AtWOX6, had a unique ortholog, suggesting the function of this clade was relatively conserved. The BpWOX2 was not homologous with AtWOX2, and was closer to the WOX3 subclade (Figure 3a). There was no unambiguous ortholog to AtWOX6 in paper mulberry in the WUS clade, as we did not obtain any sequence information of *BpWOX6* in paper mulberry. A total of nine motifs were observed from the motif elicitation tool (Supplementary Figure S12), and most of these motifs are yet to be characterized. Furthermore, we observed that most of the members in the same clade shared at least one common motif besides the homeodomain (motif 1) and the WUS box (motif 4). The ancient clade had motif 3 exclusively, while motif 2 was unique to the intermediate clade. Moreover, BpWOX8, AtWOX8 and AtWOX9 shared motif 5. Motif 6 mainly occurred in the intermediate clade and the WUS clade, and only appeared in BpWOX10 of the ancient clade at different positions. Motif 7 was in both the WOX1 and WOX4 subclades in the amino terminal. Motif 8 was only in AtWOX10 and AtWOX14, and motif 9 was specific to the WOX3 subclade (Figure 3b).

### 2.3. Transactivation Activity and Subcellular Localization of BpWOX

**Figure 4:**
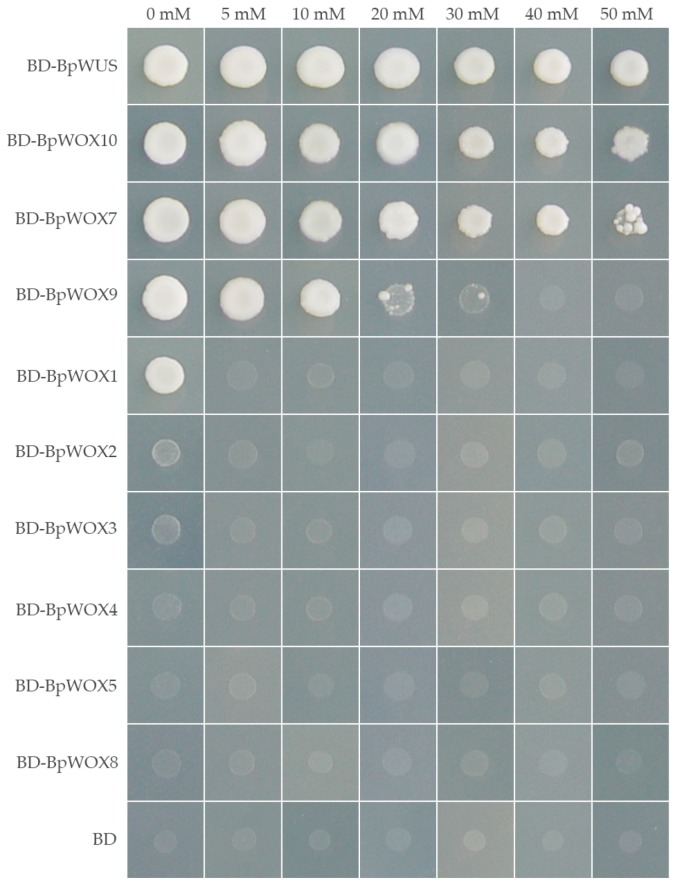
The transactivation activity of BpWOX family. The full-length ORF of each *BpWOX* gene was fused with *p*Bridge, and the transformed AH109 yeasts were selected from SD-Trp-His medium containing 0–50 mM 3-aminotriazole (3-AT), which is a competitive inhibitor of HIS3 protein. The empty *p*Bridge (BD) vector was used as a negative control.

WOX proteins function as transcription factors regulating the expression of downstream target genes. The transactivation activity of each BpWOX protein was tested using the yeast one-hybrid assay. Each of the 10 *BpWOX* genes were inserted into the yeast expression vector in a fusion of GAL4-DNA binding domain and transformed into yeast reporter cells which harbor a reporter gene, HIS3, driven by the GAL4 upstream activating sequence. The transactivation activity of each BpWOX protein was tested by the synthetic dropin medium -Trp-His (SD-Trp-His) growing experiment. The level of transactivation activity was measured by the ability of the transformed yeast cells growing on a selective medium containing 0–50 mM 3-aminotriazole (3-AT), which is a competitive inhibitor of the HIS3 protein. The results indicated that the BpWOX proteins can be classified into three groups based on their levels of transcriptional activity (Figure 4). Four BpWOXs (BpWOX7, BpWOX9, BpWOX10 and BpWUS) exhibited the highest level of transcriptional activity. BpWOX1 exhibited moderate levels of transcriptional activity as it only grew on the selective medium SD without 3-AT. Five BpWOXs (BpWOX2, BpWOX3, BpWOX4, BpWOX5 and BpWOX8) did not exhibit transcriptional activation.

**Figure 5:**
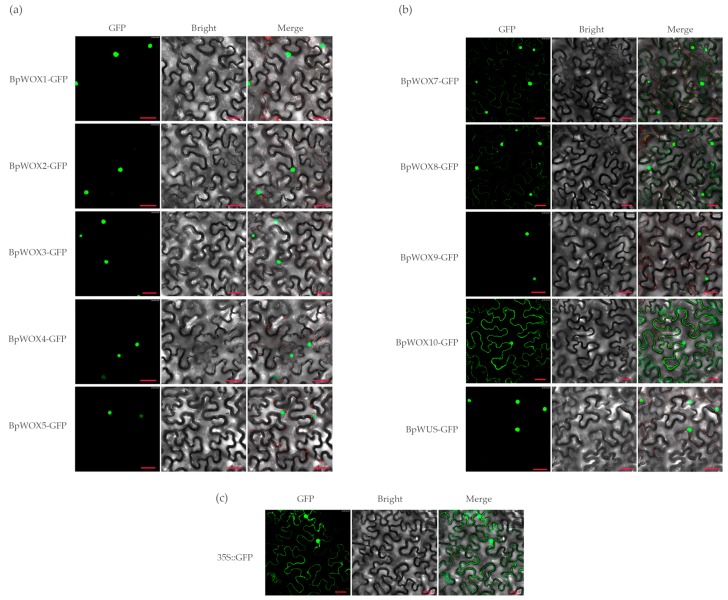
Subcellular localization of BpWOX proteins in *N. benthamiana*. Transient expression of the BpWOXs-GFP fusion protein was performed in tobacco epidermal cells. (**a**) Subcellular localization of BpWOX1 to BpWOX5; (**b**) Subcellular localization of BpWOX7 to BpWOX10 and BpWUS; (**c**) The GFP was used as a positive protein control and was detected in the nucleus and cytoplasm. Green fluorescence was observed using a confocal microscope at 48 h after *A. tumefaciens* infiltration. From left to right, the images show fluorescent-field illumination, bright-field, and overlay of three illuminations. Bar = 30 µm.

To determine the subcellular localization of BpWOX proteins, the open reading frames of each *BpWOX* gene were introduced into the *p*CAMBIA1300-GFP translational fusion construct. The recombinant *p*CAMBIA1300-BpWOXs-GFP fusions were infiltrated into the epidermal cells of *Nicotiana benthamiana*. The *p*CAMBIA1300-GFP was used as a positive protein control and was detected in the nucleus and cytoplasm (Figure 5c). The GFP signal of seven BpWOXs (BpWOX1, BpWOX2, BpWOX3, BpWOX4, BpWOX5, BpWOX9, and BpWUS) was observed exclusively in the nucleus, suggesting that these BpWOXs were nuclear proteins (Figure 5a,b). The GFP signal of BpWOX7, BpWOX8 and BpWOX10 was in both the nucleus and cytoplasm (Figure 5b), indicating that these three BpWOXs are not the nuclear localization protein. These results indicated that seven of the BpWOXs were targeted to the nucleus, and this further confirmed the function of the BpWOXs as typical transcriptional factors.

### 2.4. The Tissue Specific Expression Profiles of BpWOX Genes

**Figure 6:**
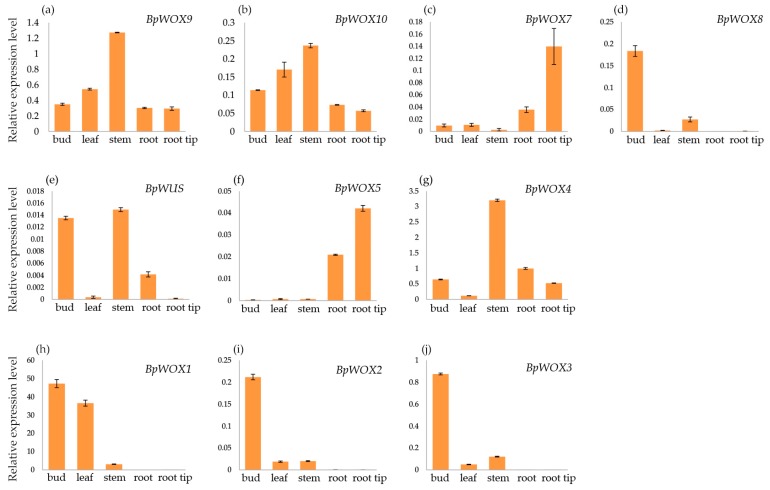
The expression patterns of *BpWOX* genes in different tissues. The expression level of *BpWOX9* (**a**); *BpWOX10* (**b**); *BpWOX7* (**c**); *BpWOX8* (**d**); *BpWUS* (**e**); *BpWOX5* (**f**); *BpWOX4* (**g**); *BpWOX1* (**h**); *BpWOX2* (**i**); and *BpWOX3* (**j**) in the apical bud, leaf, stem, root, and root tip. Transcript levels were determined by qRT-PCR. Expression of *BpGAPDH* was used as an internal control. Error bars indicate standard deviation of three independent biological replications.

To gain insights into the positions where the *BpWOX* genes were active, quantitative real time PCR (qRT-PCR) was performed for different tissues, including apical bud, leaf, stem, root, and root tip. Some *BpWOX* genes showed similar expression patterns in different tissues, such as *BpWOX9* and *BpWOX10*, which were constitutively expressed in all tissues at very high levels, implying that they may play regulatory roles at multiple development stages (Figure 6a,b). The other *BpWOX* genes were differentially expressed, suggesting that these genes have tissue specificity. *BpWOX7* was mainly expressed in the root, especially in the root tip (Figure 6c), and *BpWOX8* was mainly expressed in the apical bud and had low expression in the stem (Figure 6d). *BpWUS* was highly expressed in the apical bud and stem (Figure 6e), while *BpWOX5* was particularly expressed in the root and root tip (Figure 6f). *BpWOX4* showed a high expression level in the stem (Figure 6g), indicating that *BpWOX4* may function during the vascular development. *BpWOX1* was mainly expressed in apical bud and leaf (Figure 6h), showing that *BpWOX1* was performed in the leaf development. *BpWOX2* and *BpWOX3* had similar expression patterns, both were highly expressed in the apical bud and had low expression in the leaf and stem (Figure 6i,j), implying their functional redundancy in early leaf initiation. Compared with the ancient clade, the members in the modern clade had more specificity, indicating that the function of *BpWOX* may diverge during plant development.

### 2.5. The Response of BpWOX Genes to Environmental Stresses

**Figure 7:**
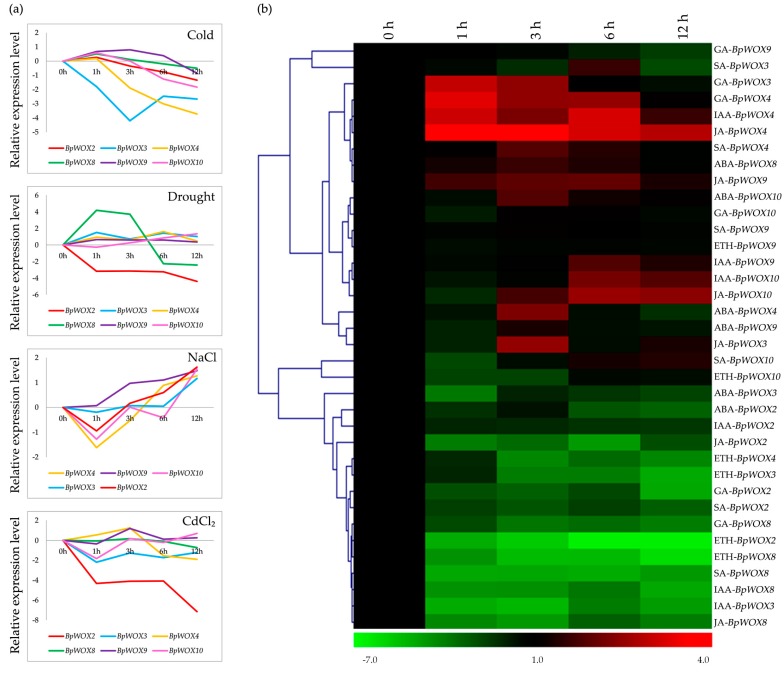
The *BpWOX* genes respond to the environmental stresses. (**a**) The expression pattern of *BpWOX* gene under four environmental conditions. For cold treatment, the seedlings were transferred into a 4 °C growth chamber. For salt and CdCl_2_ treatments, the seedlings were washed carefully and transferred into a solution of 200 mM NaCl and 50 µM CdCl_2_, respectively. For drought treatment, we used 20% (*w*/*v*) PEG solution to simulate drought condition. *BpWOX2* was labeled with a red line, *BpWOX3* was labeled with a blue line, *BpWOX4* was labeled with a yellow line, *BpWOX8* was labeled with a green line, *BpWOX9* was labeled with a purple line, and *BpWOX10* was labeled with a pink line; (**b**) A heat map of *BpWOX* genes responding to phytohormones at the transcription level. For phytohormone treatments, 100 µM solution of IAA, GA, SA, Me-JA, ETH, and ABA were sprayed onto the surface of the seedlings, respectively. Transcript levels were determined by qRT-PCR, and the expression of *BpGAPDH* was used as an internal control.

To attain a better understanding of the *BpWOX* response to various environmental stresses, we examined the dynamic expression patterns of six *BpWOX* genes under 10 different environmental stress treatments, including four abiotic stresses (cold, NaCl, drought, and CdCl_2_) and six phytohormone stresses, namely IAA, GA, salicylic acid (SA), jasmonic acid (Me-JA), ethylene (ETH), and abscisic acid (ABA), as well as used the PEG stress to imitate a drought environment. The qRT-PCR results showed that most *BpWOX* genes changed their expression levels during different stress treatments, some of them induced or repressed, and others fluctuated (Figure 7).

The expression patterns of *BpWOX* genes under four different abiotic stresses are displayed in the Figure 7a. When the seedlings were in a cold environment, almost all *BpWOX* genes were repressed in different levels across the full examination time, and four times the inhibitory effect appeared in the *BpWOX3* at 12 h and *BpWOX4* at 3 h (Figure 7a). When in a drought environment, *BpWOX8* was highly induced at 3 h and then decreased, while *BpWOX2* was repressed, and other members did not show an obvious response (Figure 7a). The responding patterns to NaCl stress were relatively consistent across all *BpWOX* genes, all of them were induced at 12 h, although *BpWOX2*, *BpWOX4* and *BpWOX10* were repressed at 1 h. (Figure 7a). Only *BpWOX2* was repressed, but other *BpWOX* genes did not show clear changes when treated with CdCl_2_ (Figure 7a).

The cluster analysis showed that most of the *BpWOX* genes were either up or down regulated by the exogenous phytohormone (Figure 7b). Overall, the expression patterns of *BpWOX9* and *BpWOX10* were induced by most phytohormones; however, *BpWOX2* was repressed by the six phytohormones (Figure 7b). The *BpWOX9* showed a high response to JA and IAA treatments, low response to the ABA treatment, but no response to GA, SA, or ETH treatments (Figure 7b). *BpWOX10* was induced by ABA, IAA, or JA treatments, and repressed by ETH treatment at 1 h and 3 h (Figure 7b). The other three *BpWOX* genes were induced or repressed by different phytohormones. *BpWOX3* was induced by GA and JA at 3 h, and repressed by ABA, IAA, and ETH across the time we examined (Figure 7b). The expression of *BpWOX8* was only increased by the ABA treatment, and decreased by the other five phytohormones (Figure 7b). In contrast, *BpWOX4* was repressed by ETH treatment, and induced by the other five phytohormones (Figure 7b). Overall, the *BpWOX* gene family responded to the various environmental stresses and phytohormones in different patterns.

## 3. Discussion

### 3.1. WOX Family Underwent Obvious Expansion in the WUS Clade

In this study, 10 *BpWOX* genes were isolated from paper mulberry, and subsequent analysis of the structure and the phylogenetic relationships further confirmed the validity of our data. Although we did not obtain any information on *BpWOX6*, the phylogenetic analysis showed that this gene may also exist in paper mulberry. Paper mulberry and mulberry are both in the Moraceae family, and have a close genetic relationship. There are 11 *WOX* members in mulberry, so we inferred that there were 11 *WOX* genes in the paper mulberry. In addition, *AtWOX6* (*PFS2*) was involved in early embryonic patterning, affecting either ovule patterning or differentiation. Due to the sterility of the paper mulberry (*B. kazinoki × B. papyrifera*), it was difficult to obtain the ovule, so we still have not isolated *BpWOX6*.

The results of the phylogenetic tree showed that WOX members of green algae and moss were only in the ancient clade (Figure 2), and the green algae are the most ancient species in the plant kingdom, suggesting that the WOX family originated from green algae. The occurrence of the vascular system (xylem and phloem) was the result of long-term adaptation of plants from aquatic to terrestrial environments. The intermediate clade contained the ancient vascular plant *S. moellendorffii* (Figure 2), implying that the evolution of the *WOX* gene was involved in vascular development. The WUS clade contained 122 members, while the intermediate clade contained 59 members, and the ancient clade contained 47 members (Supplementary Table S5). The members in the modern clade expanded rapidly, and mainly existed in the seed plants (Figure 2), where each gene in the WUS clade had a specific function in all kinds of organ. Overall, the *WOX* was a relatively ancient gene family, for the evolution of *WOX* genes might be accompanied by plant adaptation from aquatic to terrestrial environments.

### 3.2. Three of 10 BpWOX Proteins Are Typical Transcription Factors

The WOX acts as a transcription factor, which consists of four components: the DNA binding domain, *trans*-regulator domain, nuclear localization signal, and oligomerization site. The homeodomain of the WOX protein is the DNA binding domain. Previous studies have shown that AtWUS, AtWOX3, AtWOX4, and AtWOX11 [16] in *A. thaliana* were localized to the nucleus, and the PtoWUSa, PtoWOX4a, PtoWOX5a, PtoWOX11/12a and PtoWOX13 in the *Populus tomentosa* were also localized to the nucleus [33]. For the first time, we studied the transactivation activity and the subcellular localization of the WOX family as a whole in paper mulberry. According to the localization and transactivation activity, these genes can be divided into four types. The first group consisted of BpWUS, BpWOX1 and BpWOX9, which both had the transactivation activity and nuclear localization signal, suggesting that these proteins are nuclear localization protein and activate the downstream gene expression by themselves. The second group contained BpWOX2 to BpWOX5, which these four proteins are nuclear localization proteins, but did not have the transactivation activity, suggesting that they might function as transcriptional repressors. The third group was BpWOX7 and BpWOX10, which did not have a nuclear localization signal, but could strongly activate the downstream gene expression, suggesting that they may form complexes with other proteins which have the nuclear localization signal. For example, the KNOX of the homeobox super family gets into the nucleus by forming complex with BLH protein [34]. The last group only contained BpWOX8, which had neither the transactivation activity nor nuclear localization signal, and the function of this protein may not be as important as other members in the BpWOX family.

### 3.3. The Function of WOX Genes in Plant Development

We identified 10 *BpWOX* genes in paper mulberry, that is, much less than the model plant Arabidopsis, which had 15. A possible explanation may be that Arabidopsis, an annual herb, evolves much faster than the perennial wood plant. Furthermore, gene duplication and differentiation may contribute to the larger numbers of the *WOX* gene in *A. thaliana*. In general, the specific tissues where *WOX* genes are expressed may reflect their specific functions. The *WOX* genes were mainly expressed in the meristems of various organs, including SAM, RAM, and cambium, and the functions were related to either the promotion of cell division activity and/or prevention of premature cell differentiation [7]. *BpWOX9* and *BpWOX10*, which belonged to the ancient clade, were constitutively expressed at a relatively high level in the tissues examined (Figure 6a,b), indicating that they may play housekeeping functions in paper mulberry. The members in the intermediate clade had more tissue specificity when compared with the ancient clade (Figure 6c,d). *BpWOX7* was highly expressed in the root and root tip (Figure 6c), and its counterparts *AtWOX11* and *AtWOX12* were functional redundancies in the organogenesis of the adventitious root [15], suggesting that this *WOX* subclade participated in root development.

In the WUS clade, we found that some *BpWOX* genes showed similar expression patterns to their *Arabidopsis* counterparts. *AtWUS* was expressed specifically in the rib-meristem cells beneath the central zone of the SAM, which is mainly involved in stem cell maintenance. Ectopic expression of the *AtWUS* gene was shown to induce stem cells in vegetative tissues which could differentiate into somatic embryos without external phytohormones [35]. In paper mulberry, *BpWUS* was expressed substantially in apical bud, which consisted of actively dividing cells (Figure 6e). *AtWOX5* was expressed specifically in the quiescent center cells of the root stem cell niche [19], its counterpart *BpWOX5* was also highly expressed in the root (Figure 6f), indicating its function in root development. *AtWOX4* was required for promoting the proliferation of the procambial/cambial stem cells, and was expressed preferentially in the procambium and cambium [20]. In line with this, *BpWOX4* showed a high expression level in the stem, which contained plenty of vascular tissue (Figure 6g), and supported its possible role in vascular development.

The evolutionary relationship and expression pattern may have diverged between AtWOXs and BpWOXs. AtWOX7 was the only one lacking the WUS-box in the WUS clade (Figure 3b). Previous study has shown that the AtWOX7 acts as a transcriptional repressor in lateral root development. The number of lateral root primordia was increased in *AtWOX7* mutants, but decreased in plants over-expressing *AtWOX7* [36]. The phylogenetic analysis showed that the BpWOX5 was well aligned with AtWOX5 (Figure 3a), and the *BpWOX5* was definitely expressed in the root in paper mulberry (Figure 6f); however, the AtWOX7 counterpart was missed in paper mulberry. This may be the reason why the paper mulberry has a large number of lateral roots in evolution-development. AtWOX1 and AtWOX3 have functions in leaf development, especially leaf expansion and margin development, and the phenotype of single mutant or double mutant showed a narrow leaf in *A. thaliana* [23], *O. sativa* [3], *Petunia hybrida* [37], *Nicotiana tabacum* [38], and *M. truncatula* [39], suggesting these two genes have functional redundancy in leaf development. BpWOX2 was closer to the AtWOX3 subclade rather than AtWOX2 (Figure 3a), and the expression pattern of *BpWOX2* gene was similar to *BpWOX3* (Figure 6i,j). BpWOX1 was well aligned with AtWOX1 (Figure 3a), and expressed in the leaf and apical bud which contains the leaf primordium (Figure 6h). Overall, these results suggest that BpWOX1, BpWOX2 and BpWOX3 may have functional redundancy in the leaf development of paper mulberry. Taken together, the BpWOX family is involved in many processes of paper mulberry development.

### 3.4. BpWOXs Showed Divergent Expression under Environmental Stresses

As plants live in a fixed location, they need to modulate their metabolisms and change their morphogenesis to cope with new environments. Previous studies on *WOX* genes across different species have mainly focused on plant development, but their responses to environmental stresses were seldom known. Consistent with the *WOX* gene responding to the abiotic stress on rice [40], our results showed that the *BpWOX* genes did respond to environmental stresses.

**Figure 8:**
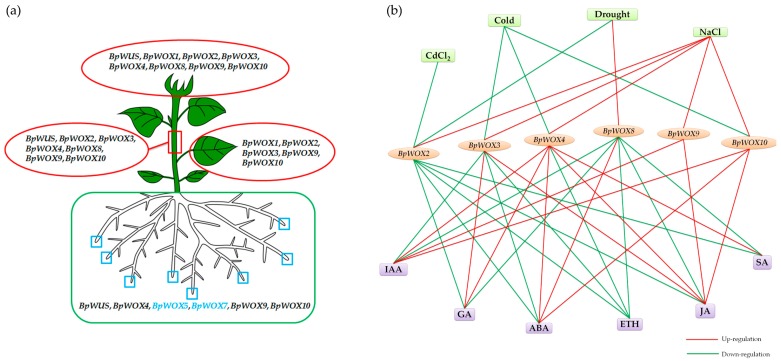
*BpWOX* genes might be involved in growth and development in paper mulberry and respond to environmental stresses. (**a**) *BpWOX* genes may participate in the tissues development and growth of the paper mulberry. *BpWOX* genes expressed in a particular tissue were enclosed together with an ellipse or box, *BpWOX5* and *BpWOX7* (blue text) were highly expressed in the root tip (blue blank box); (**b**) The network diagram between *BpWOX* genes and the environmental stresses. The line between the genes and environments demonstrates that the gene responded highly to the environment, genes that showed low response or no response were not displayed in this figure. The red line was up-regulation and green line was down-regulation.

How the *BpWOX* family is involved in organ development and responds to environmental stresses is summarized in Figure 8. The cold treatment mainly repressed the expression of *BpWOX3*, *BpWOX4* and *BpWOX10*, while the NaCl treatment induced the *BpWOX* genes expression, except for *BpWOX8*, indicating that the *BpWOX* family was sensitive to a salty and cold environment. *BpWOX2* was only induced by NaCl treatment and repressed by other treatments (Figure 8b), this was consistent with *OsWOX3*, which was highly induced by NaCl [40]; and *BpWOX2* was mainly involved in leaf development (Figure 8a), suggesting the paper mulberry might adjust the leaf morphogenesis to adapt to the hypersaline environment. CdCl_2_ stress may give the plant heavy metal ion toxicity, and all the genes were repressed in different levels, specifically, the *BpWOX2* was deeply repressed (Figure 7a), and this gene was highly expressed in the apical bud and leaf (Figure 6i), indicating the leaf of paper mulberry may receive the most injuries. It was interesting to note that *BpWOX2* was repressed by all the phytohormones, while *BpWOX4* was induced by most of the phytohormones (Figure 8b), which indicates that the same hormone concentration may function oppositely in different organs. The phytohormone abscisic acid (ABA) played a major role in adaptation to abiotic environmental stresses [41]. All examined genes were first induced then decreased by ABA treatment, and had the maximum expression at 3 h (Figure 7b), suggesting that most of the *BpWOX* genes might have the ABA response elements. In drought conditions, plants will reduce water dissipation mainly by closing the stoma, and ABA can induce stomatal closure effectively, and the expression pattern between drought and ABA treatment was similar (Figure 8b). *BpWOX2* was repressed while *BpWOX8* was induced by both these treatments, and neither of these two genes had transcriptional activation (Figure 4), but BpWOX2 protein could enter the nucleus, specially (Figure 5a), suggesting that *BpWOX2* may function opposite with *BpWOX8* in the same signaling pathway. ETH promotes plant maturation and senescence, thus when treated with ETH, most of the *BpWOX* genes were repressed except for *BpWOX9* and *BpWOX10* (Figure 8b), suggesting that the WOX gene family was a plant growth and development regulator. *BpWOX9* and *BpWOX10* were induced by other phytohormone treatments (Figure 8b), and their tissue expression patterns were similar (Figure 6a,b), suggesting their housekeeping function in the paper mulberry. Taken together, the *BpWOX* family in paper mulberry might respond to the environmental stresses by morphological adaptation via phytohormones.

## 4. Materials and Methods

### 4.1. Plant Material and Treatments

Plantlets were cultured on the MS (from Murashige and Skoog, Caisson Labs, Smithfield, UT, USA) culture media in an artificial climatic chamber at 26 °C and a 14/10 h photoperiod (day/night) for a month. For cold treatment, the seedlings were transferred into a 4 °C growth chamber. For salt and CdCl_2_ treatments, the seedlings were washed carefully and transferred into a solution of 200 mM NaCl and 50 µM CdCl_2_, respectively. For drought treatment, we used 20% (*w*/*v*) PEG solution to simulate drought condition. For phytohormone treatments, 100 µM solution of IAA, GA, SA, Me-JA, ETH and ABA were sprayed onto the surface of the seedlings, respectively. Both the control and stress treated seedlings were harvested at various periods (1, 3, 6 and 12 h), flash frozen in liquid nitrogen and stored at −80 °C for further analysis. The apical bud, stem, leaf, root and root tip of the paper mulberry subculture were collected for tissue expression analysis.

### 4.2. Isolation and the Phylogenetic Analysis of BpWOX Gene

Total RNAs were extracted with a TransZol™ RNA Extraction Kit (TransGen, Beijing, China) from each sample according to the manufacturer’s instructions. It was treated with RNase-free DNase I (Takara, Dalian, China) to remove the residual DNA. The genomic DNA of paper mulberry was extracted using a DNAsecure PlantKit (Tiangen Biotech, Beijing, China). Total RNA and DNA quality and purity were assessed with OD260/280 ratio and RNA integrity number (RIN) by using the NanoDrop 2000 (Thermo Fisher, Waltham, USA). First-strand cDNA synthesis was carried out using the PrimeScriptTM II 1st cDNA synthesis Kit (Takara) according to the manufacturer’s instructions. cDNA for amplification of the 5’ and 3’ ends of the *BpWOX* genes was prepared using a SMART™ RACE cDNA Amplification Kit (Clontech, Shiga, Japan). As the genomic sequence of paper mulberry has not been published until now, we designed the degenerate primers targeting the conserved region to obtain some fragment sequences of *BpWOX* genes. The PCR products were cloned into the pEASY-Blunt Simple vector (TransGen, Beijing) and sequenced by Majorbio (Shanghai, China). The RACE primers were designed based on the results of Sanger sequencing (Supplementary Table S2). The 5’ and 3’ ends of the *BpWOX* gene were cloned following the manufacturer’s instructions. The reaction conditions were as follows: 94 °C for 4 min; 38 cycles of 94 °C for 30 s, 68 °C for 30 s, 72 °C for 50 s; followed by 72 °C for 10 min. The primers of full length cDNA clone were designed by the sequence results of RACE-PCR products (Supplementary Table S3). The full-length cDNA sequences of *BpWOX* gene were shown in Supplementary Table S4. The genomic sequences of *BpWOX* genes were cloned using the same primers. All the PCR products were cloned into the pEASY-Blunt Simple vector (TransGen) and sequenced by Majorbio. The alignments of each *WOX* gene and the degenerate primers were displayed in Supplementary Figure S2–S11 and Table S1.

For the gene structure analysis of each *BpWOX* gene, the full length of cDNA and corresponding gDNA sequences were aligned by Muscle in MEGA 6.0 (http://en.bio-soft.net/tree/MEGA.html). The ORFs were determined by the Find ORF tool in Edit Seq 5.0 (https://www.dnastar.com/t-editseq.aspx). The MW and *p*I of putative WOX proteins were detected using the ProtParam tool (http://web.expasy.org/protparam). The 228 WOX protein sequences from 19 species were downloaded from the Plant Transcription Factor Database (PlantTFDB) (http://planttfdb.cbi.pku.edu.cn/index.php). The conserved DNA binding domain of WOX protein were aligned by Muscle in MEGA 6.0 and adjusted manually. Next, the phylogenetic tree was constructed based on the multiple sequence alignment by using the neighbor-joining method in MEGA 6.0 with 1000 bootstrap replicates. The conserved motifs of BpWOX and AtWOX proteins were observed from the motif elicitation tool (MEME, http://meme.nbcr.net/meme/).

### 4.3. qRT-PCR

First-strand cDNA synthesis was carried out using the PrimeScript RT reagent Kit (Takara) according to the manufacturer’s instruction. Real-time qRT-PCR was conducted on an MX3000PTM Real Time PCR System (Agilent Stratagene, Santa Clara, CA, USA) using the SYBR-Green PrimeScript RT-PCR Kit (Takara). Each reaction was carried out with a volume of 20 µL, which contained 10 µL PCR Master Mix containing SYBR, 0.4 µL ROX, 6.8 µL ddH_2_O, 2 µL diluted template and 0.4 µL of each of two gene specific primers. Three technical replicates were taken in each biological replicate. The *BpGAPDH* gene was used as an internal control, transcript levels were normalized against the average expression of the *BpGAPDH* gene. The 2^−ΔΔ*C*t^ method was used to analyze the data. Information about the primers used in qRT-PCR can be found in Supplementary Table S8.

### 4.4. Subcellular Localization of BpWOX Proteins

The cDNA encoding the ORF of *BpWOXs* was digested with suitable restriction enzymes and inserted into the *p*CAMBIA1300-GFP expression vector to generate the BpWOX-GFP fusion protein under the control of the cauliflower mosaic virus (CaMV) 35S promoter. The restriction enzymes and primers used to construct the recombinant expression vectors for subcellular localization are listed in Supplementary Table S9. The recombinant *p*CAMBIA1300-BpWOX-GFP plasmid was transferred to the *A. tumefaciens* strain EHA105 and introduced into the *N. benthamiana* epidermal cell. After 48 h of normal cultivation, fluorescence was examined by fluorescence microscopy (Leica TCS SP5, Wetzlar, Germany).

### 4.5. Transactivation Activity Assay

The cDNA encoding of the ORF of *BpWOXs* was digested with suitable restriction enzymes and then cloned into the *p*Bridge vector to yield a fusion protein in frame with the GAL4 DNA binding domain. The restriction enzymes and primers used to construct the recombinant expression vectors for transactivation activity assay are listed in Supplementary Table S9. After sequence analysis, the recombinant *p*Bridge-BpWOXs were transferred to the AH109 yeast strain. Transformed yeasts were cultured on SD medium without His and Trp. Then yeast cells were then dropped on SD-Trp-His plates containing various concentrations (0 to 50 mM) of 3-AT to test their activities. The plates were incubated at 30 °C for 3–5 days before photographing.

## 5. Conclusions

In this study, 10 *BpWOX* genes were isolated from paper mulberry by RACE-PCR. The phylogenetic analysis showed that the 10 BpWOXs belonged to three classical clades. Most of the BpWOXs (except BpWOX7, BpWOX8, and BpWOX10) were localized to the nucleus, and only five BpWOXs (BpWOX1, BpWOX7, BpWOX9, BpWOX10 and BpWUS) possessed transcriptional transactivation ability in the yeast one-hybrid system. Our results showed the expression level of *BpWOX* genes across different tissues was related to their evolution relationships, from the ancient clade to the WUS clade, and expression was organ-specific. The *BpWOX* genes responded to a range of environmental signals, and these results contribute to our understanding of the morphogenesis of paper mulberry under adverse environmental conditions. Thus, future investigation into the specific function of BpWOX might provide further insights into the leaf blade expansion and shape diversity in paper mulberry. The results presented here will be helpful for future study on the biological functions of BpWOX proteins.

## Figures and Tables

**Table 1:** Sequence information of *BpWOX* family in paper mulberry.

Gene Name	GenBank Accession	gDNA Length (bp)	cDNA Length (bp)	ORF Length (bp)	AA Numbers	MW (Da)	Isoelectric Point (*p*I)
*BpWUS*	MF420354	2931	1275	900	299	32518	6.14
*BpWOX1*	MF420355	2861	1728	1212	403	45811	6.87
*BpWOX2*	MF420356	3241	1439	1095	364	41653	9.69
*BpWOX3*	MF420357	1925	1088	723	240	27653	8.7
*BpWOX4*	MF420358	1463	1105	744	247	27949	9.44
*BpWOX5*	MF420359	927	805	624	207	24082	7.32
*BpWOX7*	MF420360	3055	1119	858	285	31261	6.4
*BpWOX8*	MF420361	2756	1639	1260	419	45929	8.26
*BpWOX9*	MF420362	2920	1397	816	271	31020	6.23
*BpWOX10*	MF420363	2393	1256	780	259	29625	5.13

bp: Base pair; ORF: open reading frame; AA: amino acid; MW: molecular weight; Da: dalton; pI: isoelectric point.

## Supplementary Materials

Supplementary materials can be found at www.mdpi.com/1422-0067/18/8/1782/s1.

## Abbreviations

HOX	Homeobox
WOX	WUSCHEL-related homeobox
HD	Homeodomain
RACE	Rapid amplification of cDNA ends
qRT-PCR	Quantitative real time PCR
ORF	Open reading frame
UTR	Untranslated regions
aa	Amino acid
MW	Molecular weight
NJ	Neighbor-Joining
3-AT	3-Aminotriazole
Cd	Cadmium
QC	Quiescent center
SAM	Shoot apical meristem
PRS	Pressed flower
GA	Gibberellin
IAA	Indole-3-acetic acid
ABA	Abscisic acid
JA	Jasmonic acid
SA	Salicylic acid
ETH	Ethylene
ERF	Ethylene response factor
EAR	ERF-associated amphiphilic repression
