# Supplementary material for: Identification and Functional Divergence Analysis of WOX Gene Family in Paper Mulberry
Source: Int J Mol Sci. 2017 Aug 16;18(8):1782. doi: 10.3390/ijms18081782 (PMC5578171; doi:10.3390/ijms18081782)
Supplement: Supplementary file 1 [file ijms-18-01782-s001.zip › ijms-212119-supplementary-v6.pdf]

Supplementary Materials: Identification and Functional Divergence Analysis of *WOX* Gene Family in Paper Mulberry

Feng Tang, Naizhi Chen, Meiling Zhao, Yucheng Wang, Ruiping He, Xianjun Peng and Shihua Shen

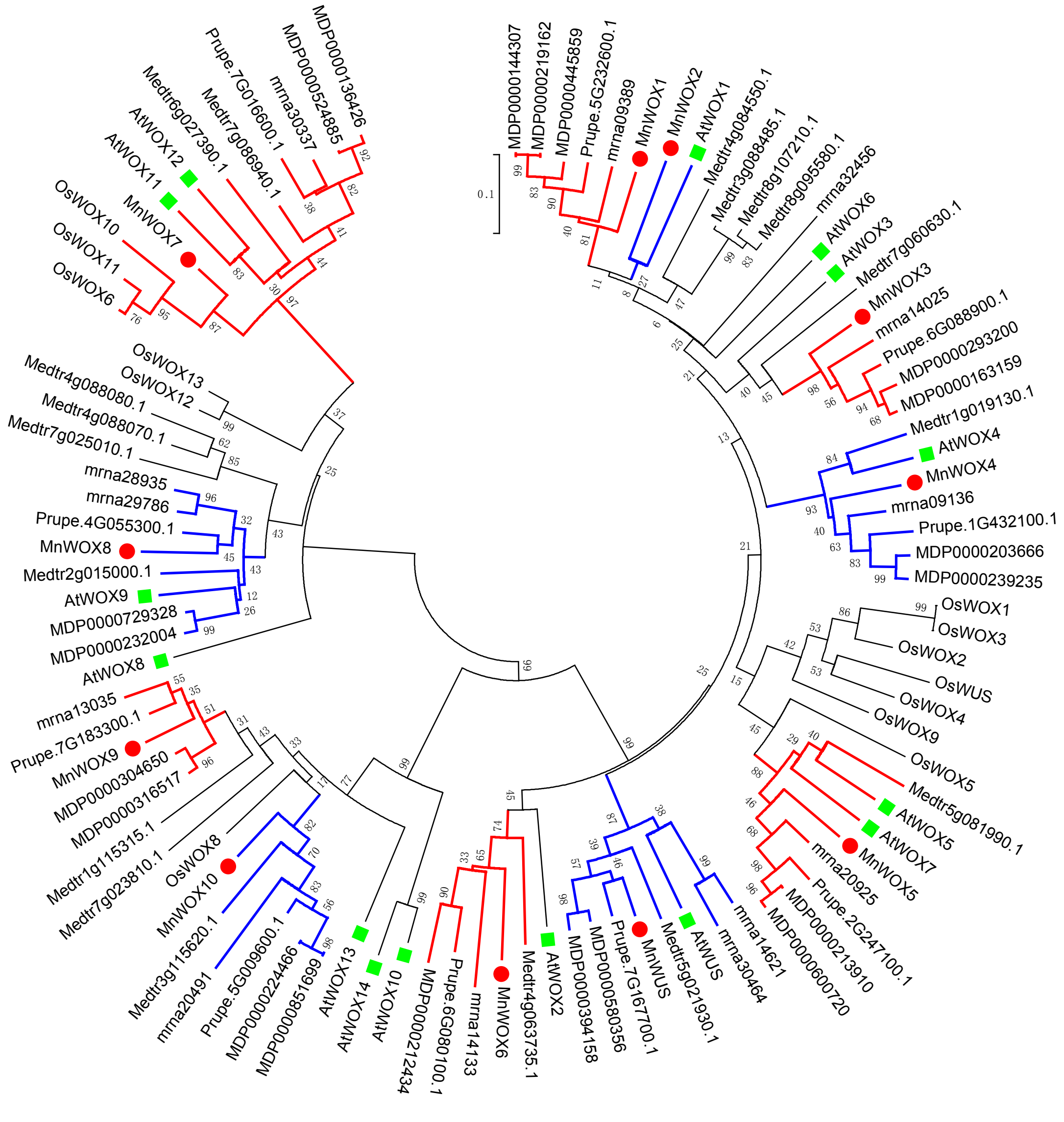

Figure S1. Phylogenetic analysis of *BpWOX* genes in *Morus notabilis*, *Fragaria vesca*, *Prunus persica*, *Malus domestica*, *Medicago truncatula*, *Arabidopsis thaliana* and *Oryza sativa*. There were eleven *WOX* genes in mulberry. Thus, we infered that there might be eleven *WOX* genes in paper mulberry. Each of subclade *WOX* gene from seven species were aligned to find the conserved region and design the degenerate primers. The selected sequences for alignment were labeled with red or blue line. The MnWOX proteins were labeled by red dots, and the AtWOX proteins were labeled by green square. *M. notabilis*, Mn; *F. vesca*, mrna; *P. persica*, Prupe; *M. domestica*, MDP; *M. truncatula*, Medtr; *A. thaliana*, At; *O. sativa*, Os.

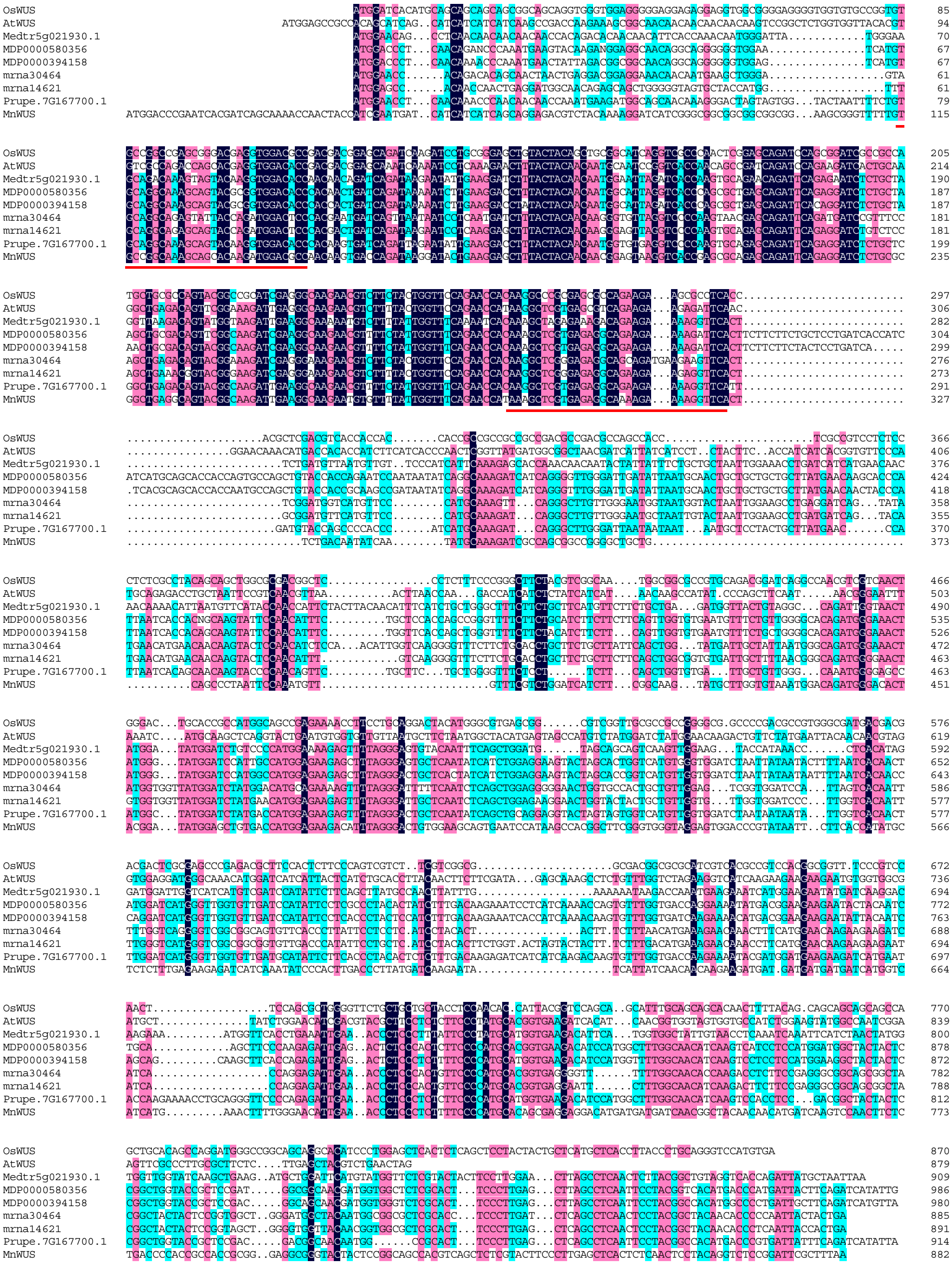

Figure S2. The sequences of *WUS* subclade were aligned to find the conserved regions and design the degenerate primers. The red line was the location of degenerate primers for *BpWUS*.

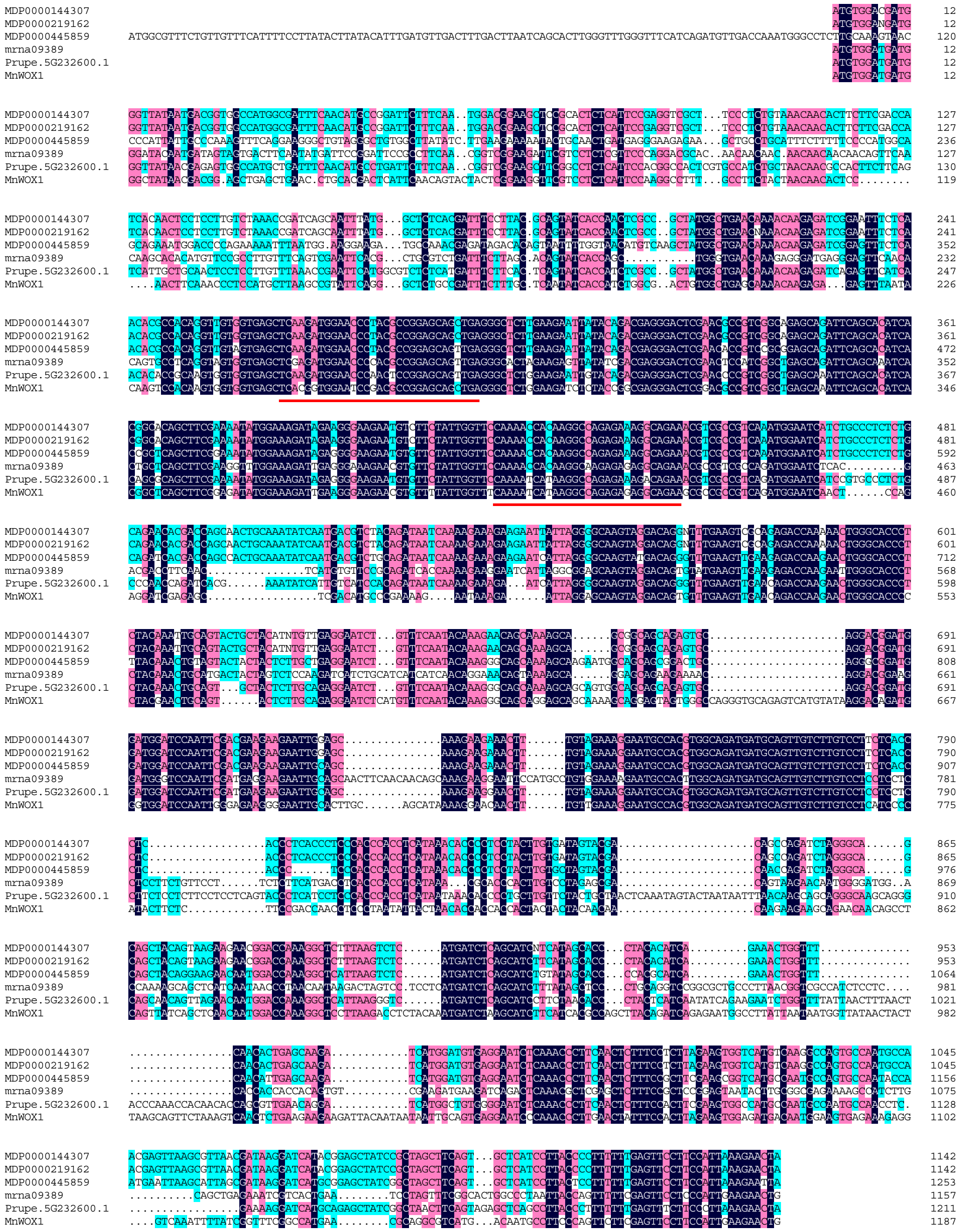

Figure S3. The sequences of *WOX1* subclade were aligned to find the conserved regions and design the degenerate primers. The red line was the location of degenerate primers for *BpWOX1*.

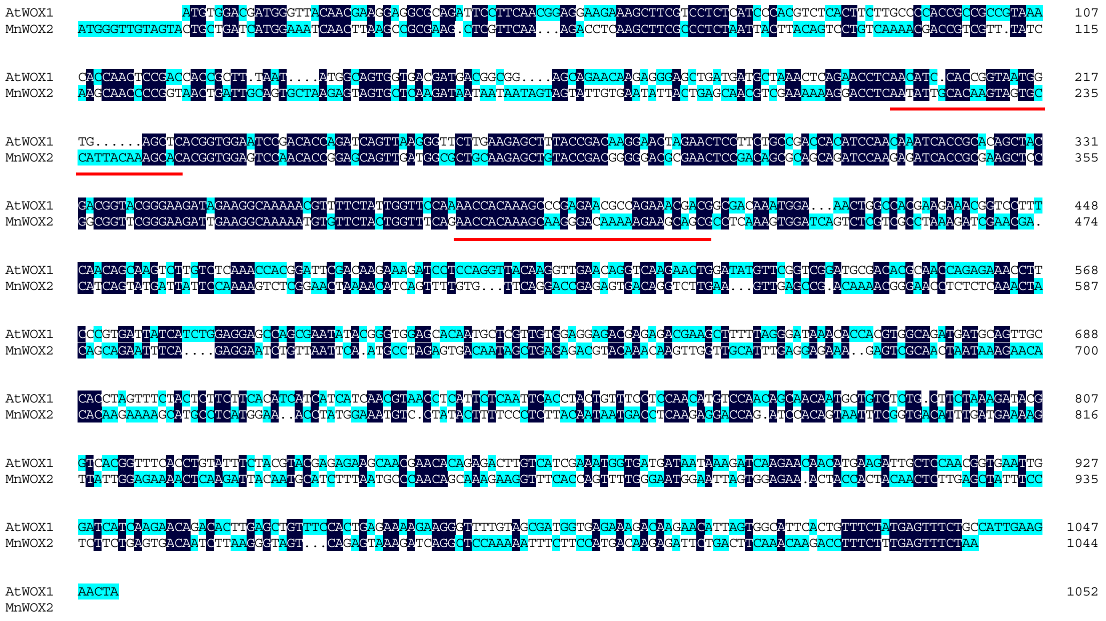

Figure S4. The sequences of *WOX2* subclade were aligned to find the conserved regions and design the degenerate primers. The red line was the location of degenerate primers for *BpWOX2*.

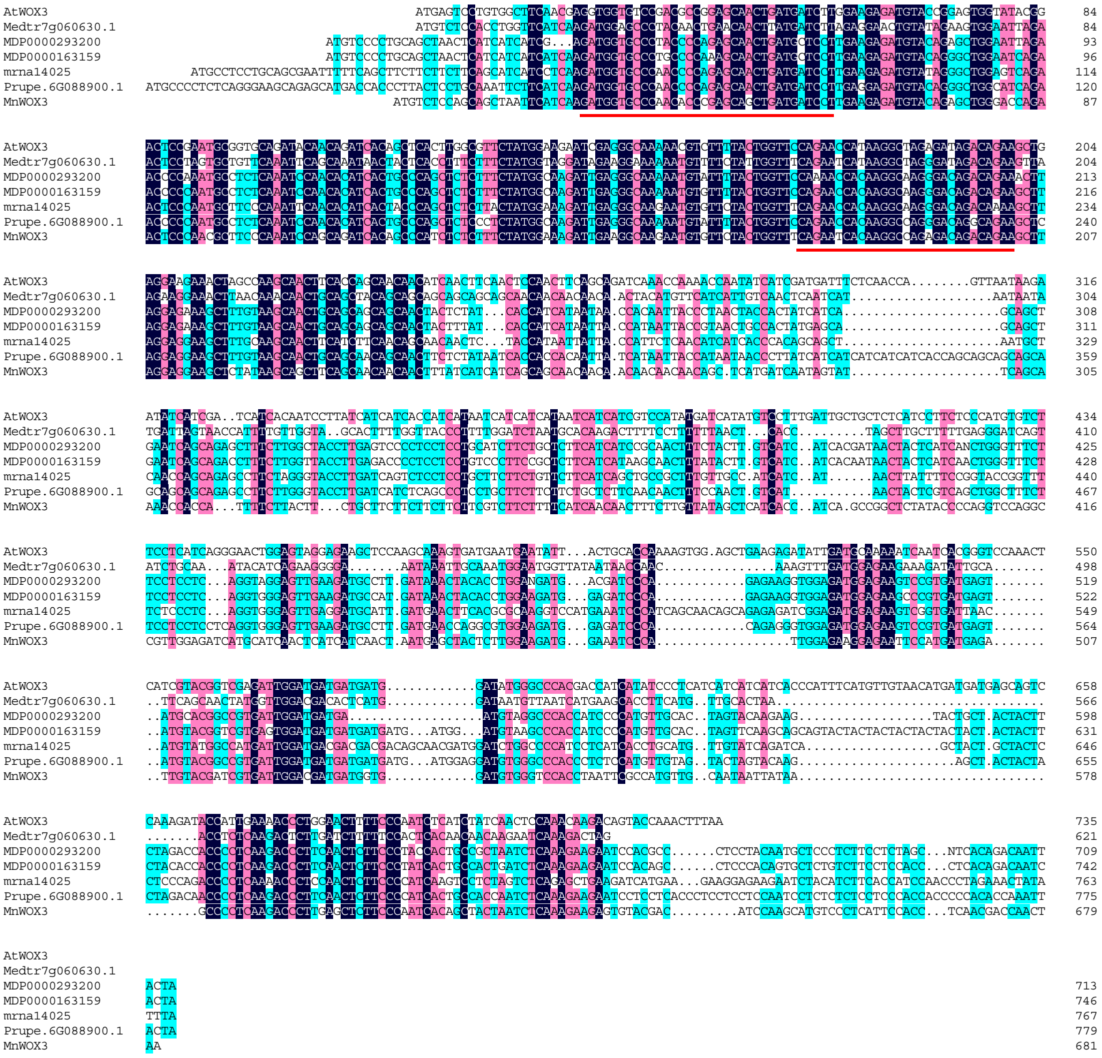

Figure S5. The sequences of *WOX3* subclade were aligned to find the conserved regions and design the degenerate primers. The red line was the location of degenerate primers for *BpWOX3*.

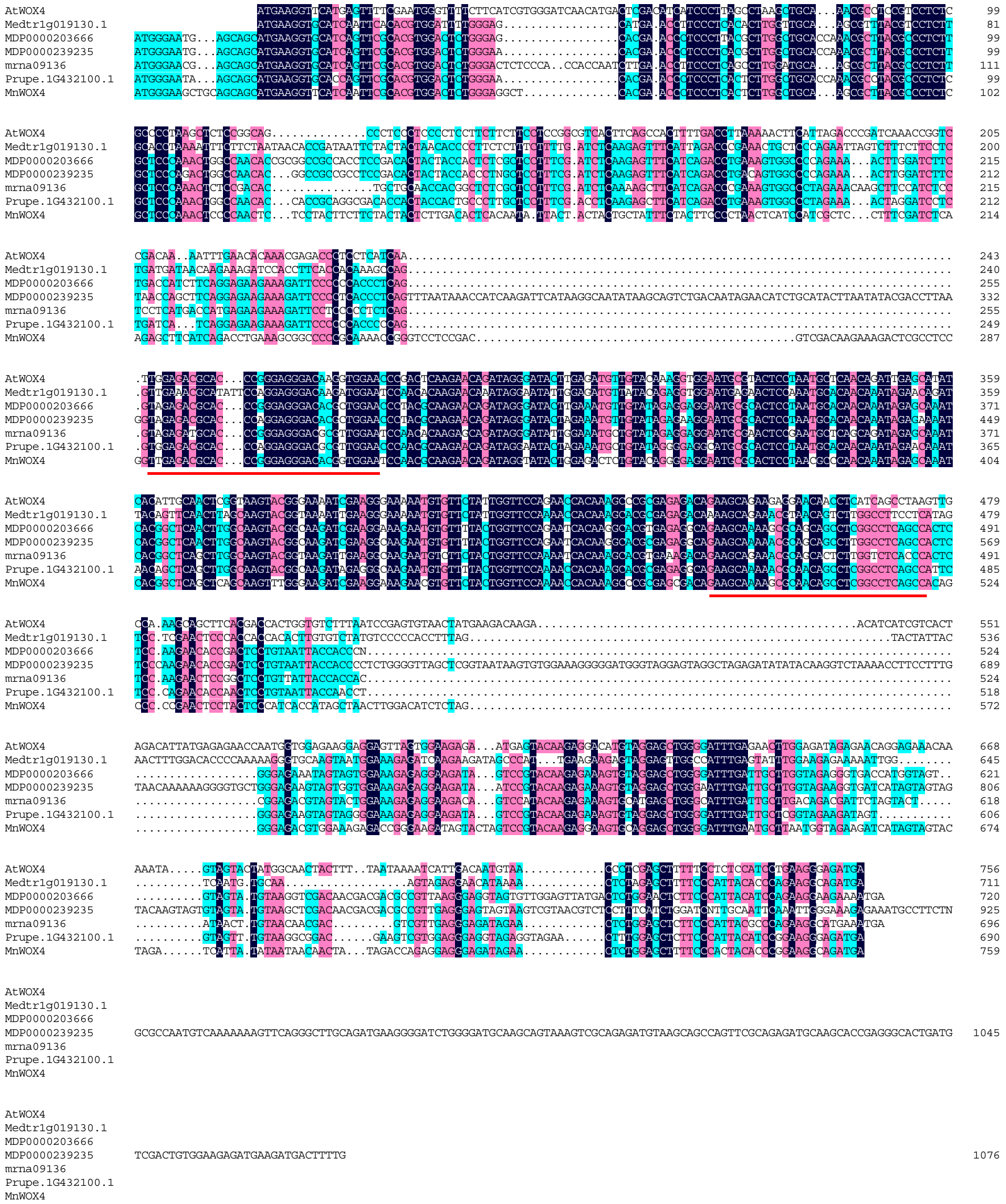

Figure S6. The sequences of *WOX4* subclade were aligned to find the conserved regions and design the degenerate primers. The red line was the location of degenerate primers for *BpWOX4*.

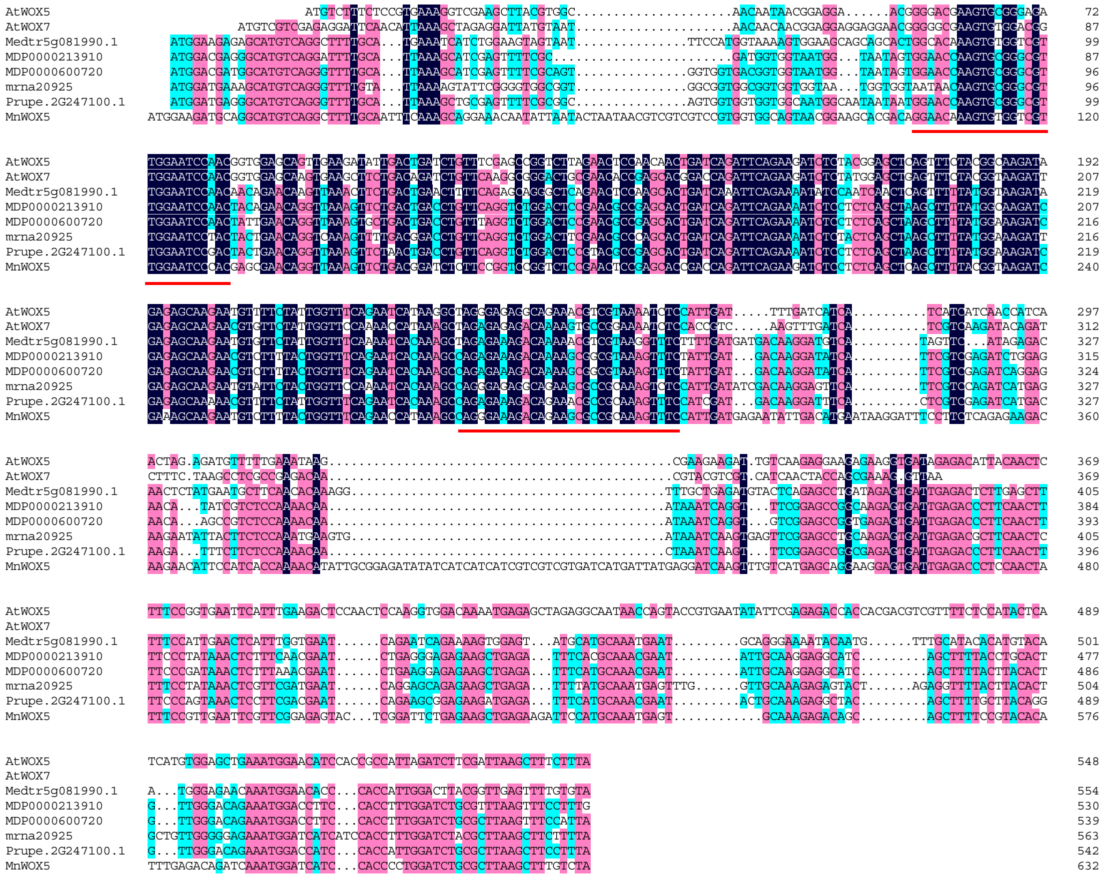

Figure S7. The sequences of *WOX5* subclade were aligned to find the conserved regions and design the degenerate primers. The red line was the location of degenerate primers for *BpWOX5*.

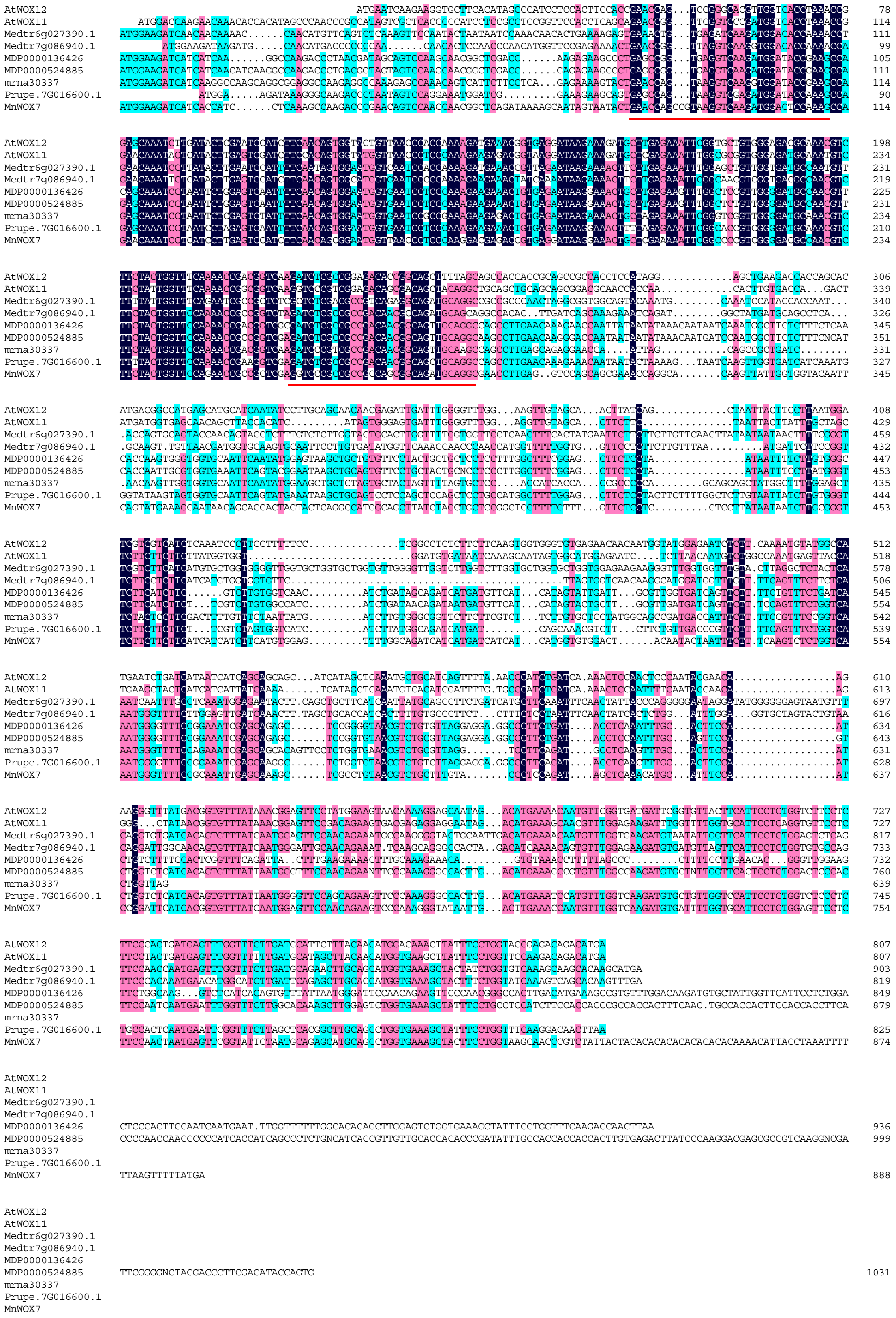

Figure S8. The sequences of *WOX7* subclade were aligned to find the conserved regions and design the degenerate primers. The red line was the location of degenerate primers for *BpWOX7*.

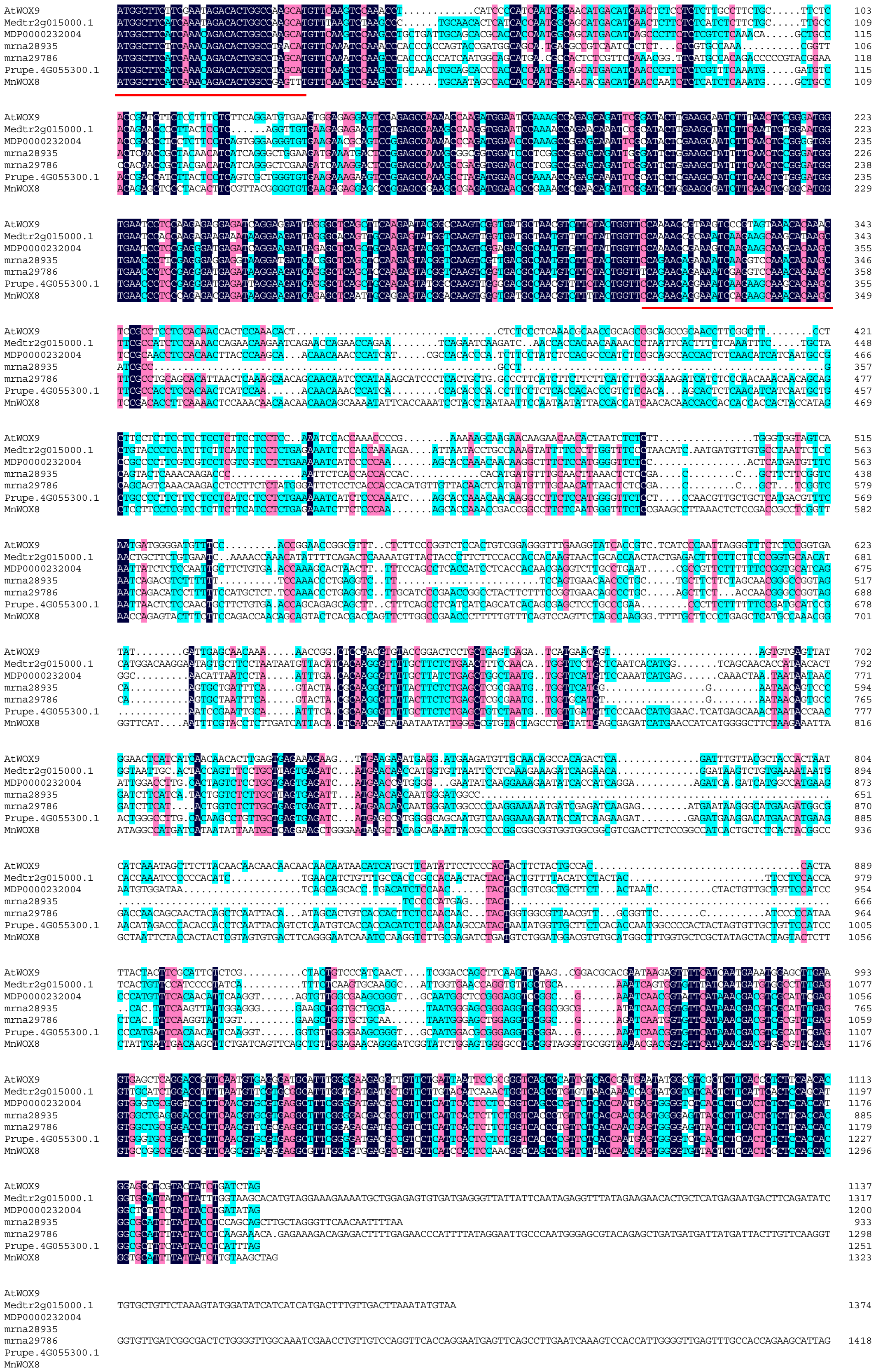

Figure S9. The sequences of *WOX8* subclade were aligned to find the conserved regions and design the degenerate primers. The red line was the location of degenerate primers for *BpWOX8*.

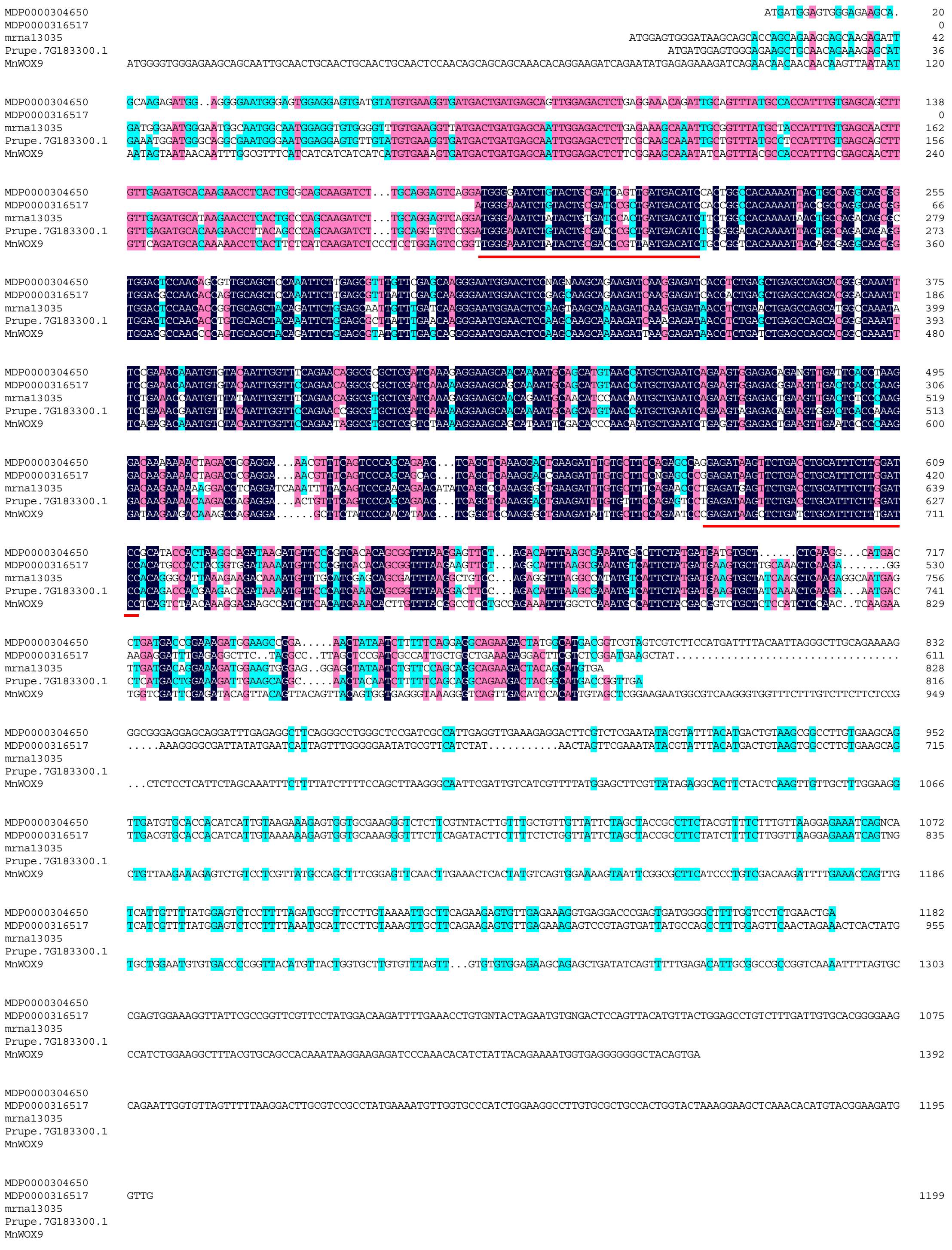

Figure S10. The sequences of *WOX9* subclade were aligned to find the conserved regions and design the degenerate primers. The red line was the location of degenerate primers for *BpWOX9*.

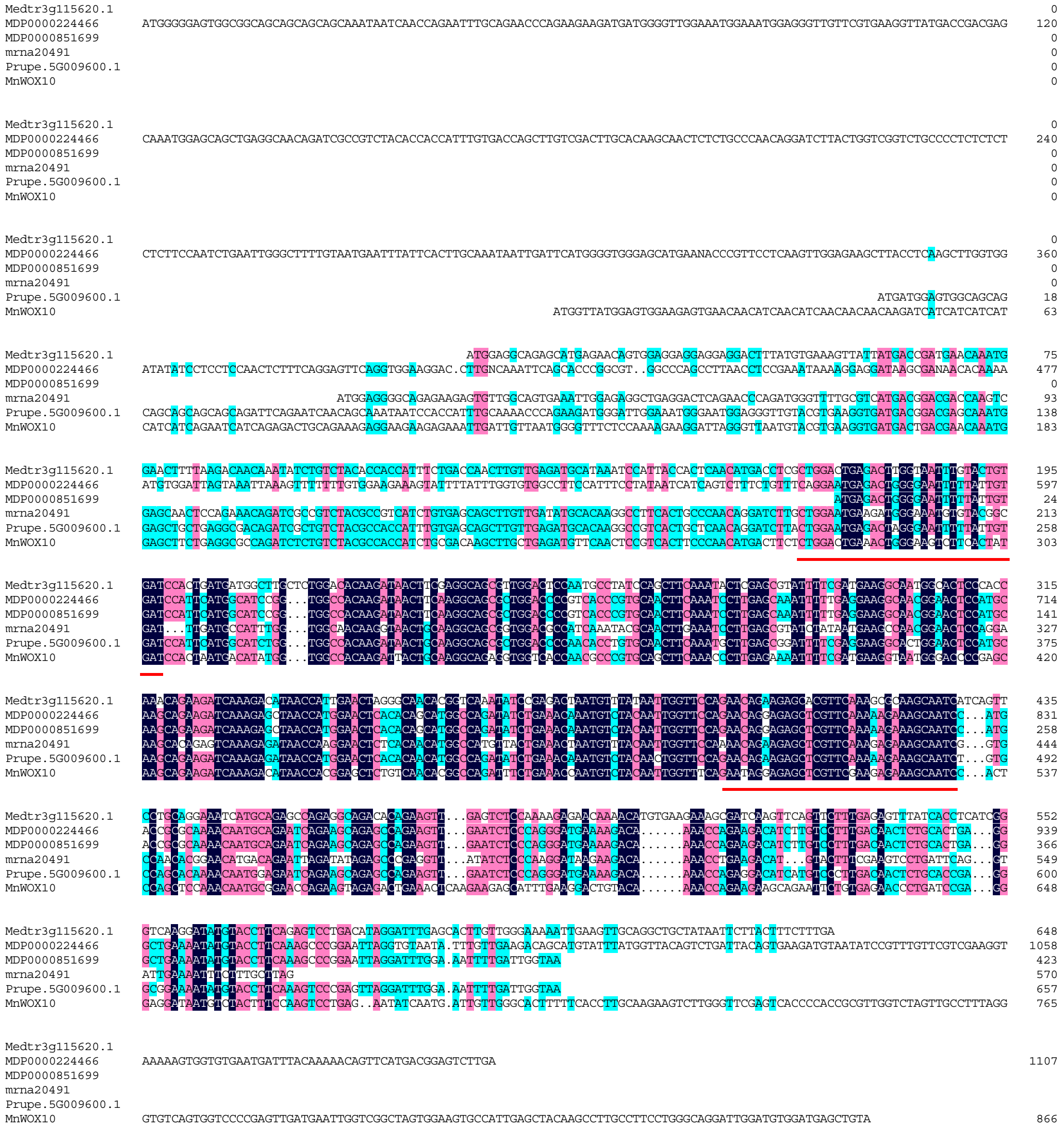

Figure S11. The sequences of *WOX10* subclade were aligned to find the conserved regions and design the degenerate primers. The red line was the location of degenerate primers for *BpWOX10*.

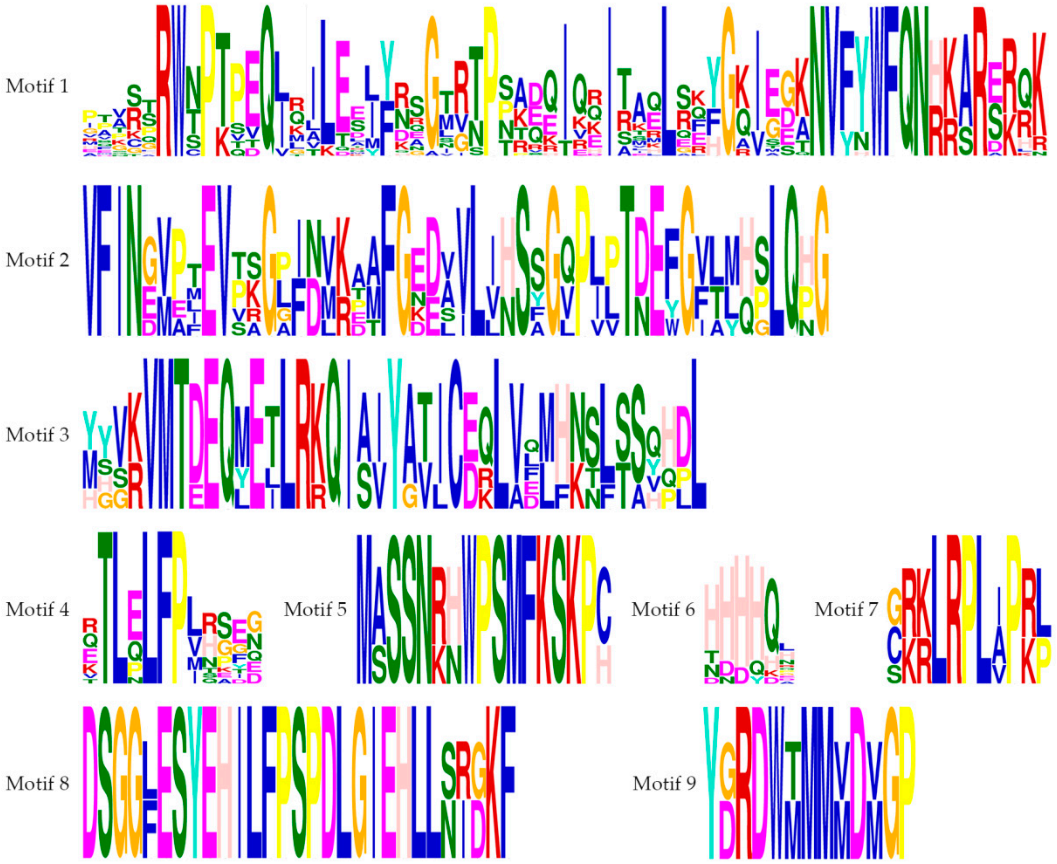

Figure S12. The conserved amino acid sequences of WOX protein in *A. thaliana* and *B. papyrifera*.

Table S1. The degenerate primer sequences of *BpWOXs* used to get fragments from paper mulberry.

Gene Name	Forward primer (5′-3′)	Reverse primer (5′-3′)
BpWUS	TGCMGGCAAAGCAGYACAAGRTGGACDCC	TGAACCTTTTCTTYTGCCTCTCNCGAGCYTT
BpWOX1	TCAAGATGGAACCCNACDGAGCAGYTGAGG	GYTTCTGCCTYTCTCTGGCCTTRTGRTTTTG
BpWOX2	AATATTGCACAAGTAGTGCCATTACAAAGCAC	CGCTGCTTCTTTTTGTCCCTTGCTTTGTGGTT
BpWOX3	GATGGTGCCCAACVCCCGAGCARCTGATGMTC	TTCTGYCTRTCYCTDGCCTTGTGRTTYTG
BpWOX4	GTDGAGACGCACCCRGGAGGGACRMGNTGGAA	GGCTGAGGCCRAGGCTGYTGCGYTTYTGCTT
BpWOX5	GGAACVAAGTGYGGDCGTTGGAATCCNAC	GARACYTTGCGGCGCTTCTGYCTYTCYCTG
BpWOX7	GAACCGNGCCGTNAGGTCAAGDTGGAYWCCAAAG	GCCTGCADCTGCCTBTGDCGGCGDCGRGABC
BpWOX8	ATGGCTTCWTCAAACAGACACTGGCCDAGCAT	GCTTGTGYTTGCTTCTBGATTTNCKGTTYTG
BpWOX9	TGGGAAATCTRTACTGCGAYACGYTGATGACATC	GGATCMAAGAAATGCAGRTCAGARCTYATCTC
BpWOX10	CTGGAMTGARRCTDGGDARTBTTCACTATGAT	GATTGCTTTCTYTTYGAACGAGCTCTYCTGTT

Table S2. The sequences of PCR products amplified with the degenerate primers in paper mulberry. The fragment sequences are used to design the RACE-PCR primers. The red backgrounds are the 3′-RACE primers, and the green backgrounds are the 5′-RACE primers.

Gene Name	Sequence (5′-3′)
BpWUS	TGCAGGCAAAGCAGCACAAGATGGACACCAACAAGTGACCAGATAAGGATTCTCAAGGACCTTTACTACAACAATGGCGTGAGGTCCCCAAGTGCAGAGCAGATTCAGAGGATCTCTGCCCGGCTGAGGCAGTACGGCAAGATTGAAGGCAAGAATGTCTTTTATTGGTTTCAGAACCATAAAGCTCGCGAGAGGCAAAACAAAAGGTTCA
BpWOX1	GGTGGAACCCGACGCCGGAGCAGCTGAGGGCCCTCGAAGAGCTCTACCGCCGCGGGACCCGAACGCCATCGGCCGAGCAAATTCAGCACATCACGGCTCAGCTTCGGAGATATGGAAAGATTGAAGGGAAGAACGTGTTCTATTGGTTCCAAAATCACAAGGCCAGAGAGAGGCAGAAGC
BpWOX2	AATATTGCACAAGTGGTGCCATTTCAAAGCACGCGGTGGAGTCCGACGCCGGAGCAGTTGATGGCGCTGCAAGAGCTGTACCAGCGTGGGACGCGGACCCCAACGGCGCAGCAGATCCAGGAAATCACCGCGAAGCTCCGGCGGTTCGGGAAGATTGAAGGCAAAAACGTGTTCTACTGGTTTCAGAACCACAAAGCCAGGGACAAAAAGAAGCAGCG
BpWOX3	TCAAGATGGTGCCCAACACCCGAGCAGCTGATGATCCTGGAAGAGATGTACAGGGCTGGAACAAGAACTCCCAACGCGTCTCAAATCCAACAGATCACAGCCCATCTCTCGTTCTACGGAAAGATTGAAGGCAAGAATGTGTTCTACTGGTTTCAGAACCACAAGGCCAGGGACAGGCAGAAGC
BpWOX4	GTTGAGACGCACCCGGGAGGGACGCGGTGGAATCCGACGCAAGAACAGATAGGGATACTGGAGACGCTGTACAGAGGAGGAATGCGCACTCCGAACGCGCAACAAATAGAGCAAATCACGGCTCAGCTGGGCAAGTTCGGCAAGATCGAAGGCAAGAACGTGTTCTACTGGTTCCAGAACCACAAGGCCCGCGAGCGGCAGAAGCAGAAGCGCAACAGCCTCGGCCTCAGCC
BpWOX5	GGAACAAAGTGCGGCCGTTGGAATCCCACGAGCGAGCAGGTTAAAGTCCTGACCGATCTCTTCCGGTCCGGTCTCCGAACCCCGAGCACCGACCAGATCCAGAAGATCTCCGCTCAGCTCAGCTTTTACGGCAAGATCGAGAGCAAGAACGTCTTCTACTGGTTTCAGAACCACAAGGCCAGAGAGAGACAGAAGCGCCGCAAGGTCTC
BpWOX7	GAACCCGCCGTGAGGTCAAGGTGGACTCCAAAGCCAGAACAAATTCTCATCCTTGAGTCCATCTTCAATAGTGGAATGGTAAACCCTCCCAAGGACGAGACCGTGAGGATACGGAAGCTACTCGAGAAATTCGGGGCCGTTGGCGACGCCAACGTATTCTACTGGTTCCAAAACCGCCGCTCGAGGTCCCGCCGTCGCCAGAGGCAGATGCAGGC
BpWOX8	ATGGCTTCATCAAACAGACACTGGCCCAGCATGTTCAAGTCCAAGCCTTGCAACACCCACCCCCACCAATGGCAACACGACATCAACCAGTCTCTCATCTCAACTGGCTGCCACAGAACTCCCTACACTTCAGTTGGCGGGTGCGAGGAGCGGAGCCCGGAGCCGAAGCCGAGATGGAATCCGAAGCCGGAGCAGATTCGGATCCTGGAAGCGATCTTCAACTCGGGGATGGTAAACCCGCCGAGAGACGAGATAAGAAAGATCAGGGCACAATTGCAGGAGTACGGCCAAGTGGGTGACGCCAACGTGTTTTACTGGTTCCAGAACAGGAAATCCAGAAGCAAACACAAGC
BpWOX9	TGGGAAATCTATACTGCGACACGTTGATGACATCTGCGGGCCACAAGATCACGGCGAGACAGCGTTGGACTCCGACTCCTGTGCAGCTACAGATTCTGGAGAGCATGTTTGACCAGGGGAATGGAACTCCAAGCAAGCAAAAGATCAAAGAGATAACCTCTGAACTGAGC

	CAGCATGGCCAGATTTCGGAGACTAATGTCTACAATTGGTTCCAGAACAGGCGTGCT CGGTCGAAAAGGAAACAGCAGAATAATGCAGCACCCAACAATACTGAATCTGAAGT GGAGACTGAAGTTGAGTCACCTAAGGACAAGAAGACAAAGCCGGAGGAGTTTCTGT CCCAACAGAACTCGGCTTCGAGGGCTGAAGATATCTGCTTCCAGAGCTCCGAGATCA GCTCTGACCTGCATTTCTTTGATCC
BpWOX10	CTGGAATGAAGCTGGGAAGTCTTCACTATGATCCACTAATGACATATGGTGGCCACAA GATCACTGCAAGGCAAAGGTGGTCACCAACGCCTGTGCAGCTTCAAACTCTAGAGA AAATTTTCGATGAAGGGAATGGGACTCCGAGCAAGCAGAAGATCAAAGAGATAACC GTAGAGCTGAGTCAACATGGCCAAATTTCGGAAACCAATGTCTACAATTGGTTCCAG AACAGGAGAGCTCGTTCGAAGAGAAAGCAATC

Table S3. The primer sequences of *BpWOXs* used for full-length cDNA amplifications.

Gene Name	Forward primer (5′-3′)	Reverse primer (5′-3′)
BpWUS	ACATGGGGACCATCCCACATAACATTTT	GTGTCAAGACCATCGTCATATATAATAATCA
BpWOX1	GATAATAGTACTACACTACAATGCCTACCTCC	CCATTTTAAGGCAAACCCTCCTTTTTCA
BpWOX2	ATGTCCATTTGAAGCCTAGATATCAAACTA	AAATCTTCTATATTTTACTCACGTCCTTTATTC
BpWOX3	ACCCCAAGAGACAAAGACAAAAGTCGAATC	AGTCTTTTGAAGGTAAACAAGGCATTTC
BpWOX4	CCTCACCTCATCATTTGGTCTTGGTTATC	TGATCTGCTGCTTATTGGAACCAAAGAGCG
BpWOX5	ATACATGCACACGGGTTTCACATTG	TCATAAACATAAACTACCGATGTCCTACCGTAG
BpWOX7	CAATGGAAGATCATCACCATCATCAAGGC	GTTAAAGACGTAATATTTATGGGAGACTGGC
BpWOX8	ATCTCATCAGTACCAAAAACAAGAGCAAC	CCACGAAAAGCATTCATTTTTACTACAC
BpWOX9	GTTTGTCTCTTTCTTTCCAACCTTTTTC	GGTTCATCCATTCATATCCAATCATAAAATG
BpWOX10	AGTGTCAGTACTAAACACTTGCTTGCAATCTAAG	GGTTTTTCAATAGACCCAGTTCATCATAATCTG

Table S4. The full-length cDNA sequences of *BpWOX* genes. The gray backgrounds are the ORFs.

Gene Name	Sequence (5′-3′)
BpWUS	ACATGGGGACCATCCCACATAACATTTTCACTTTCTCTCTCCAAATTCCCTCTCTCTCTCTCT<br>CTCTCTCTCTCTCTCTCTCTCTCTCTCTCTCTCTCTCTCTCTCTCTCTCTCTCTCTCTCTTGTTTGCT<br>TCCTCTTTGTAATCTTCTTGTCTAATCTCACCATGGAACCTCAACAACAAAACCAATCAGCACC<br>AAATGACCATGAAAATGGCGGCAACAAAGGAGGGTCGTTTCTTTGCAGGCAAAGCAGCACA<br>AGATGGACACCAACAAGTGACCAGATAAGGATTCTCAAGGACCTTTACTACAACAATGGCGT<br>GAGGTCCCCAAGTGCAGAGCAGATTCAGAGGATCTCTGCCCGGCTGAGGCAGTACGGCAAG<br>ATTGAAGGCAAGAATGTCTTTTATTGGTTTCAGAACCATAAAGCTCGCGAGAGGCAAAACAA<br>AAGGTTCACTTCTGACATGCAAAGACCTCCTCCCCCTGCTTCTTCTAATGGCCTAATTCCAAAT<br>AATGCTACTTCTACTACTAATAATATTCATCAATCCTTTCACCCGCACCACAAGTACTCCAACAT<br>ACCTCCTGCAGATTTTGCAGTTGTTCCATCAGGATCTCCTTCTTCGGCAAATATTGCTTGGTGTT<br>AATGGACAGATGGGGCACTATGGATATGGAGCTATGACCATGGAGAAGACATTTAGGCTGCA<br>GGACTGTGGAAGCAGTGGATCCATAAGCCACAACTTCGGGTGGGTCGGAGTGGACCCGTATT<br>CTTCACCGTACGCTCTCTTTGAGAAGAGATCACCAATCCCATATGACCCTTATGATCAAGATGA<br>TCATGATGAACACGAGGATCATGATCATCAAGAAACTGGAGGGAACATTGAAACCCTCCCAC<br>TCTTCCCCATGCACGGCGAGGAGGACATCATGATGATCAACGGCTACGGCAACATGATCAAG<br>CCCGGCTTCGCCGACCCCACCGCCACCGGAGGTGGCTACTACTCCGGTGGCCACGTCAGCTC<br>TCGCACTTCCCTTGAGCTAACCCTCAACTCCTACGGGTCACCGGATTCGCTTTAAAAGCATTTC<br>TAAACCCTTTTCAAGTTAATCACGTGTCAAATGTTAATTAACTCCTTAATCACTCCCATGCATGA<br>AACATGGTGGATTAAATTAATTAGCTCAGCTAGCTACTTATAATTAATCATGGACGTTGATTAGT<br>TATGTTATATTGTTACATGCTCTATTTATGATGATTGTGTGATTATTATATATGACGATGGTCTTGA<br>CAC
BpWOX1	GCTCTCTCTCTCTCTCTCTCTCTAGATAATAGTACTACACTACAATGCCTACCTCCGAATGTCTC<br>AAAACTAAATCATCACTTCACTGTTTCAGCTCCAACTAACCCCATTTTTCAACTCTAGCTAGCT<br>TCAATAGTATCTCAATACTATACAGCCTTCTAAATACAAGCTCTATCCACTTTCTATTTCCCTTGC<br>ATGTTCTGATCTATGTCCACTTTCGTAACTCGAAACAGTATCCAAACACCACTCTGAACCCTAT<br>CATATCATAAAACACAGTAGCCCATTACGAAAGAAAAAAGAGAGTTAGAGAGAAATCCTAGA<br>AAAAAAAAAAAAAACATGTGGATGATGGGCTATAACGACGGAGGTGAACTGAATCTTCACG<br>ACTCTTTCAACAGCACTTCTCGTAAGCTTCGTCCTCTCATTCCAAGACCTTTGCCTTCTTCTAAC<br>AACGCCCCAACCTCAAACCCTCCATGTCTAAGCCGTATTCATGCTTCTGCTGATTTCTTTGCTC<br>AATATCACCATCTGGCTACTGTGGCTGAGCAAAACAAGAGAGAGTTCAATACAAGTCCGCAA<br>GTGGTGGTGAGCTCGCGGTGGAACCCGACGCCGGAGCAGCTGAGGGCCCTCGAAGAGCTCT<br>ACCGCCGCGGGACCCGAACGCCATCGGCCGAGCAAATTCAGCACATCACGGCTCAGCTTCGG<br>AGATATGGAAAGATTGAAGGGAAGAACGTGTTCTATTGGTTCCAAAATCACAAGGCCAGAGA<br>GAGGCAGAAGCGCCGCCGTCAGATGGAGTCGGCTCCAGACGAGACCGACATGTCCGAAAAG<br>AACAAAGAATTAGGGGCAAGTAGGACAGTGTTTGAAGTTGAACAGACCAAGAACTGGGCAC<br>CCCCTACTAGCTGCAGTACTCTTGCAGAGGAATCTCATGTTTCAATACAAAGGGCAGCAGCAG<br>CAGCAAAAGCAAGAGTGGGCCAGGCTGCAGAGTCATGTATGAGGACAGATGGTTGGATCCA<br>ATTGGGCGAAGGAGAATTGAATTTGCAGCAATATAGAAGGAACAACTTTGTGGAAAGGAATG<br>CCACGTGGCAGATGATGCAGTTATCTTGTCCTCATCCCCATGCTCAGCCCACCAACCTCATAAA<br>CACCCCTTGTAATATTAATAACAACAATAATATTATTAGTAATACCACCACCAGTAGTACTACTA

	CCACTACCACTACAGCTAGAACAACAGCATCAACAATGGACCAACAAAGGTTCATGAAGAC<br>CTCTACACATGATCTAAGCATCTTCATCACGCCGGCCTACAGATCAGAAAATGGCCTTATGAAT<br>AACATTGGTTATAACCACTTTAACAGTTCTAATGTCAACTCTGAAGAAGAAGATTACAACAAT<br>TGCAGTGAGGAATCTCAAACCCTAGAACTGTTTCCGCTTAGAAGTGGCGATTACAACGGAAG<br>CGAGAAAGAGGCTCATGATCGATTATCAGTTGCGGACATGAACGCCGCGGGCATCATGACCA<br>CTCCTTTCCAGTTCTTTGAGTTCCTACCATTGAAGAATTGATCATGTGTTGATCAACAAAAGTTT<br>GTAATCTCATTGGAAAACGAGACCTTTTTTAAAATTTAATTTAGTTTTTTTTTCCCCTTTTCTT<br>TTCTTGTTTTTGTTTCGATGTTTACATGTCGTGGAATTGGTGACTATGGTATATGTGGGAATGAA<br>AAAGGAGGGTTTGCCTTAAAATGG
BpWOX2	ATGTCCATTTGAAGCCTAGATATCAAACTATCATTTCACTTTCTTCTTTCCCTCCTTGTTACAATA<br>TCCTCCTCTACCCATCTCTTTGCTCAAAAACAGAATTCCTCTGTGAGAGAGAGCATTTTATACT<br>ATATAGAGCACTGTCTACAATGTGGACGATGGGTCGTAGTACTACTGATCATGGAAATGAAGTT<br>AAGATTCCTAGTTCCTTCAAAGACCTCAAACTTCGGCCTATAATTATTTACAGCCCTTTCAAAA<br>CGACTGTCGTTTCTCCAACAACTCCGGTCACCGATTTCAGTGCTAAGAGTACTCAAGATAATA<br>GTATTAATAGTAATATTGTGAATACCGAGCAACGTCGACGAAGAAAGGACCTGAATAATAATA<br>TTGCACAAGTGGTGCCATTTCAAAGCACGCGGTGGAGTCCGACGCCGGAGCAGTTGATGGCG<br>CTGCAAGAGCTGTACCAGCGTGGGACGCGGACCCCAACGGCGCAGCAGATCCAGGAAATCA<br>CCGCGAAGCTCCGGCGGTTCGGGAAGATTGAAGGCAAAAACGTGTTCTACTGGTTTCAGAAC<br>CACAAAGCCAGGGACAAAAAGAAGCAGCGACTCAAAATGGAGTCTTATAATAATCGCTCCA<br>AGAATATTGATTATAATTCCAAAAGCCAGGTTGAAACGAAAACATCAGTTTTGTCCTTGGGAC<br>CGAGAGTGACAGGGCTTGAATATGAGCAGACAAAACGGGAACCTCTCTCAAACTACAGCAG<br>AATTTCAGAGGAATCTGTTAATTCAATACCTAGAGTGACAACTACAACAACAGCAGAGACATC<br>AGGTACAAACAATAAGTTGGTTGCATTGGAGGAGAAAGAGAAGTCACAACTACTAACAACA<br>AACAACATTTTTCAAGAAAAGCAAAATGCCTCGTGGAAACCTATGGAAATGTCCTACTCTTTT<br>CCCACTTACAATAATTATGAGCTCAAGAGGACCAGTCCCACAGTAATTTCGTTGACATTTGATG<br>AGAATTTAATGGTGAAAAAGCAAGATTACAGTGCCAGTGTATCTTTAATGCCCAACAGCAAAC<br>CAAGTTTCACCAGTTTTGGGAATGGAGTCAATGGCGAAAAAGAAAGTACTGCTACAACTCTT<br>GAGCTATTTCCTCTTCGGAGTGACAATCTAAAGGGTGCTCAGAGAACAGAAATTCATTCCGGC<br>ACAAAAGATTCCGATTTCAAACCAGACCTCTCGTTGCGTTTCTTCCTCTGAAGAACTGACTCG<br>AGCATTAGAGGACTAGTGATTGGGCCTTCCAACAGAAATAAAAAGTGTAGTGATTTTTATAAG<br>TTTCTTTTATTTTGGTTTGGTTAGTTTTCTGTGTAATTTAATGTTTTCTTTTCGTGTCATGCATGGA<br>CTGACATTAATGCAAAGTAAGAATAAAGGACGTGAGTAAAATATAGAAGATTT
BpWOX3	ACCCCAAGAGACAAAGACAAAAGTCGAATCTGATCTTTTCCCAAATCGAGTTTGCTCAACAT<br>CATTGCACATTAAAAAAAAAAAAGAAAAAATCATCAATAAAATGTCTCCAGCAGCCAATTCA<br>TCAAGATGGTGCCCAACACCCGAGCAGCTGATGATCCTGGAAGAGATGTACAGGGCTGGAAC<br>AAGAACTCCCAACGCGTCTCAAATCCAACAGATCACAGCCCATCTCTCGTTCTACGGAAAGA<br>TTGAAGGCAAGAATGTGTTCTACTGGTTTCAGAACCACAAGGCCAGGGACAGGCAGAAGCTC<br>AGGAGGAAGCTCTACAAGCAGCTTCAGCAGCAACAGCTCTATCATCAGCAGCAGCAGCAAC<br>AGCAGCAGCTAATGATCAACAGTGCTATTCAGCAAAACCACCATTTTCTTGGCTATTTTGACTC<br>TAGTACTCCTAATAGTTCTGCTTCTTCTTCTTCTTCTTCTTTGCATCATCAGCAGCTTTGTTGTTAT<br>AACTCATCATCACCATCAGCTGGTTCTCTACCCCAGGTCCAGGGTGGAGTTGAAGATCATCAT<br>CATGCATCAACTCATCATCATCAACTGATGGGCTACACTTGGAAGATGGAAATTCCCCTGCTTG<br>AGAAGGAGAACTCCATGATGAGAATGTACGATCGTGATTGGACGATGATGGTGGACGTGGGC<br>CCACCTAGTTCTCCATATTGTTGCAATAAACCCCTCAAGACCCTTGAGCTCTTCCCCATCACCG

	CTACTAATCTCAAAGAAGAGTGTACGACGTCAAAACATGTCCCTCATTCCACCCCAACGATGA<br>CCATCTAATGACCAGTCTAAATTCCTATCTGCATGGCTCTCTTTCTCTCTCTCTCTCTCTCTCT<br>CTCTCTCTCTCTAAGTCTATGTCTAGATGCTACAAGGGTAGGCTTGAAAGTGTCTGTGTGTTGTT<br>TATTTTCTTGGTTTCTGTTATTTGTCTAGAGCTCTAGGAAGAGAAAGATCGCAATTGTCTAGCGA<br>GTAATACGTTACTGGCTCTATTGTCCTATTACTACACCTACTCAATGAAATGCCTTGTTTACCTTC<br>AAAAGACT
BpWOX4	CCTCACCTCATCATTTGGTCTTGGTTATCCCAAGCACCACCTCATTTCCCACTTTGGTGACTTTA<br>ATCGTACCAGTACATTACTTTGCTATATAACCAGGGGTTTGTGTAGTGATTCAGTCCTCCACCAT<br>GGGAAGAAGTAGCTGCAGCATGAAGGTGCATCAGTTCGCACGTGGACTCTGGGAGGCTCACG<br>AACCCTCCCTCACGCTTGGCTGCAAACGCTTACGCCCTCTTGCTCCCAAGCTCCCTAACTCTCC<br>TCCTTCTACTACCGACACTACTCACATTACTACCACTGCTTCTTCCCCCCACTCCATCGCTCCTT<br>TCGATCTCAAGAGCTTCATCAGACCCGAAAGCGGGCCCCGAAAGCTCGGTTCCTCCGACGAC<br>AAGAAAGACTCGCCTCCGGTTGAGACGCACCCGGGAGGGACGCGGTGGAATCCGACGCAAG<br>AACAGATAGGGATACTGGAGACGCTGTACAGAGGAGGAATGCGCACTCCGAACGCGCAACA<br>AATAGAGCAAATCACGGCTCAGCTGGGCAAGTTCGGCAAGATCGAAGGCAAGAACGTGTTCT<br>ACTGGTTCCAGAACCACAAGGCCCGCGAGCGGCAGAAGCAGAAGCGCAACAGCCTCGGCCT<br>CAGCCACAGCCCGCGAACCCCGACCCCAATCGCCATAGCCACCTGCGGTTTGGACACTAGGG<br>GAGACTTGGACAGAGACCGGGAAGATAGTACTAGTCCGTACAAGAGGAAGTGCCGGAGCTG<br>GGGATTTGAATGCTTGGTAGAAGAGCATAGTAGTAGAATATTGTATAACAATTATAGAGATCAG<br>GATCAGGGAGATAGGACTCTGGAGCTTTTCCCATTACACCCGGAAGGCATGAGATGATGGGG<br>GGTTTTGGGGAGAGAGAAAATACTAGTAATAATTCATCCTTCTTTCACCATCATATCATTTTTCA<br>GCTTTAGTTTATGTGGCCGGTATATGAATATTATCAGCTTAATATGTGCTTTTCTTTCCTTTTTCTT<br>CTTTTTTTCTTTCGCTTCGGGAAAAGAAGAAAAAAGAAGGTACTTGTCTGATCTCTTTTGTAGT<br>ACTGATCTGCTGCTTATTGGAACCAAAGAGCG
BpWOX5	GCACACGGGTTTCACATTGAAAACTGATCAGAAACTGCTATATCACTAGGAAACTTCAGTGAC<br>AGAGATCAGAAGATTTTGGAGCTCAAAAAATGGAAGATCATGGCATGTCAGGCTTTTGCATTA<br>AAGCGGGACATAACATGGCTAACAACGTTGTCGTTCGCGGTACTACTGGAACCAACGGCAGC<br>ACTGGAACAAAGTGCGGCCGTTGGAATCCCACGAGCGAGCAGGTTAAAGTCCTGACCGATCT<br>CTTCCGGTCCGGTCTCCGAACCCCGAGCACCGACCAGATCCAGAAGATCTCCGCTCAGCTCA<br>GCTTTTACGGCAAGATCGAGAGCAAGAACGTCTTCTACTGGTTTCAGAACCACAAGGCCAGA<br>GAGAGACAGAAGCGCCGCAAGGTCTCCGTTGATGATCATGACAAGGATTTCGTTCTAAGAGA<br>GGACAAGATTCCCTCTCCCAAACGTAATTTTGCGGAGATATATCACAGTCGCCATCATCATCAT<br>CATCATCATCATCATGATCACGAGGAATATCAGGTTTCTGAGCCGGAAAGAGTGATTGAGACG<br>CTCCAACTCTTTCCGTTGAATTCGTTCGGAGAGCCGGATTCGGAGAAGCTGAGATTCCATGAT<br>CAGTGCAGAGAAACAGCGGCTTTTCCGTACACAATTGAGTCAGCAATAGAACATCCTCCAGT<br>ACTGGATCTGCGCTTAAGCTTTGTCTAATTAAGCATGTTTAGTTTACATCTTAGCCCAAAAAAA<br>AAGAAAGAAAGAAAAAGAGAGAGATAGGTTTTTGTCATCACGTATTAATTAGG
BpWOX7	CAATGGAAGATCATCACCATCATCAAGGCCAAGACGCTAACAGTCCAAGCAACGGCTCCGAG<br>AAAAGTAGTAGTGCTAGTACCGAACCCGCCGTGAGGTCAAGGTGGACTCCAAAGCCAGAAC<br>AAATTCTCATCCTTGAGTCCATCTTCAATAGTGGAATGGTAAACCCTCCCAAGGACGAGACCG<br>TGAGGATACGGAAGCTACTCGAGAAATTCGGGGCCGTTGGCGACGCCAACGTATTCTACTGGT<br>TCCAAAACCGCCGCTCGAGGTCCCGCCGTCGCCAGAGGCAGATGCAGGCCAGCCTCGAAGT<br>CCAGCAGCGGAACCAGGCCGCACAAGGTGTTGGTGGTGCATTTCAGTATGAAAGCAATAATT<br>ATAACAGCAATAGTACTACTCAGGCCATGGCCGGTTATGTAGCTGGTTCGGCTTCTTTTGTTTG

	CTCTCCTTCTTATAGTAATCTTGTGGGTTCTTCTTCTTCCTCTTCATCATCTTCATGCGGAGTTTTG<br>GCAGATCATCATGATCACCATCATGGTTTGGACTACAATACCAATTTCTTTCAAGTCTCTGGTC<br>AAATGGGTTTTCCGCAAATTGAGCAAAGCTCTCCTGTAACGTCTGTTTTGTATCCTCCAGATAG<br>CTCAAACATGCACTTCGAATCTGGATTCATCACAGTGTTCATCAATGGGGTTCCAACAGAAGT<br>TCCTAAGGGCCTAATTGACTTGAAACCAATGTTTGGAAAAGATGTGATTTTGGTGCATTCCTCC<br>GGAGTTCCACTTCCAACTAATGAATTTGGCATTCTAATGCAGGGCTTGCAGCCTGGTGAAAGC<br>TATTTCCTGGTGCCGAGAACAACGTTAAGTAATAACTGAAGCAGCATCTAGCTAGATTGCTAC<br>CAATGGAAGCTTCCCTACATATTCCAATCTTGATTTCTTCATTCCTTTTTTTCTCTCCTTATACATT<br>TATTATAGATTTCTATTTTGTGGTTTTAAGGATTTAAATTGAGAGTGCTATTATATATATGTGTGTG<br>ATTATTAGAGACAATTAAGATCTATACATAAGAGTTGCCAATATCATGCATATGATTGCTTATTT<br>GTAATGCCAGTCTCCCATAAATATTACGTCTTTAAC
BpWOX8	ATCTCATCAGTACCAAAAACAAGAGCAACAAAACGGCTACTTCATCTCATTGTTTCTCTTTTGT<br>TTATCTACTTTAAATTACTTCTCAGATCATCAGTTTCATCATCATCATCATCATGGCTTCATCAAA<br>CAGACACTGGCCCAGCATGTTCAAGTCCAAGCCTTGCAACACCCACCCCCACCAATGGCAAC<br>ACGACATCAACCAGTCTCTCATCTCAACTGGCTGCCACAGAACTCCCTACACTTCAGTTGGCG<br>GGTGCGAGGAGCGGAGCCCGGAGCCGAAGCCGAGATGGAATCCGAAGCCGGAGCAGATTCG<br>GATCCTGGAAGCGATCTTCAACTCGGGGATGGTAAACCCGCCGAGAGACGAGATAAGAAAG<br>ATCAGGGCACAATTGCAGGAGTACGGCCAAGTGGGTGACGCCAACGTGTTTTACTGGTTCCA<br>GAACAGGAAATCCAGAAGCAAACACAAGCTCCGACACCTCCAAAACTCCAAACAACAACA<br>ACAACAGCAAACTCAGAACAATCACCAAAACTCTCCGAACTCCAATTTTACCACCACTACAA<br>CCACTAGTAGTACTACTACTCCTACCACCAGCACAAGAGCTCCTTCCTCATCGTCCTCGTCTTC<br>CTCGGAGAAATCGTCTCCGAAAGCCCCGAACAGATCGCCGATCTTCTCGATGGGCTTCACCG<br>AAGCCCTAAACTCTCCGACCGGCTCGGTCAACCAGAGCACCTTCTTTCAGACCAACGGTAGT<br>ACCATCACTAGTACTGGTCACGACCCGTTCTTGGCCGAACCCTTTTTCTTCCCGGTGCAACAGT<br>CCAGTACTAGTACTACTCAAGGGTTTTGCTTCCCTCATGATCAGCTCACGCCGACTGCGGTCCA<br>TCAGATTATGCCCGCAGATCATTATCAACAGATTAACAACATCGGACCGTGCACTAGCCTCTTG<br>CTCAGCGAGATCATGAACCATCATGGGGCTTCAACGAAAGGCCATGATCATGATCATCATAAG<br>CTGCTACCGCTTAATTACACTCCGTTGGAGGCGGCGGCGACGACGGTGGCAAGGACTACTTCT<br>CCGGCCAGTACTCCTCTCACTACGGCTGCTACTAGTACTATTGAATCTTCAACATCACAAAGGA<br>ATCAAATCCAAGCAGGTGTTGGAGAACAGGGAACTTCTGGAGTGGGGCCAATCGGAGGGGG<br>CGGTAAAACGACGGTGTTCATAAACGACGTGGCGTTTGAGGTGCCGGCGGGGCCGTTCAATG<br>TGAGGGAGGCGTTTGGGGAGGAGGCTGTGCTCATCCACTCCTACGGTCAGCCAGTTCTCACC<br>AACGAGTGGGGGGTCACTCTTCACTCCCTCCAGCCTGGTGCTTTTTATTATCTAATTTAGGAGC<br>TTGGGAGATATATCTAGCTAGATATGAGAAAAACACACGCTGGCTGTGGTTCATTTAATTCACT<br>ACATATATTTCTGCTCTTATCTCTTTAAGTTTGGATTAGGGCTTTCAATGACTTGTTATATTAGCTC<br>TGTTAATTAGTCCGTTTAGTGACAACTTGAAAATTTGGTGGACTCATATTATATATATGTATTCTC<br>TTTCAGTTGTTAAGTAGAGAATTATATCAAAGATGTGTAGTAAAAATGAATGCTTTTCGTGG
BpWOX9	GTTTGTCTCTTTCTTTCCAACCTTTTTCCTGTCTCTCTCTCCTCTCTCCGACTAAAGACTGGTTTT<br>TTAGACAAACATACTCCTCCGTTTCCCAGACTGTGAGCCTGAGAAAGAGGAGAGAGAAAGA<br>AGAGAGAGAGAGAGAGAGAGAGAGAGAGAGAGAGGGAGGATGATGGGGTGGGAGAAGCAGG<br>AGGAGAGAGAAGGGCATAATAATAGTAACAGCAGTAATCTGTATCATCACCATGTGAAGGTG<br>ATGACTGACGAGCAATTGGAGACGCTTCGCAAGCAAATCTCCATCTACGCCACCATTTGCGAG<br>CAGCTCGTTCAGATGCACAAGAACCTCACTTCCCATCACGATCTCCCTGGCGTCCGATTGGGA<br>AATCTATACTGCGACACGTTGATGACATCTGCGGGCCACAAGATCACGGCGAGACAGCGTTG

	GACTCCGACTCCTGTGCAGCTACAGATTCTGGAGAGCATGTTTGACCAGGGGAATGGAACTC<br>CAAGCAAGCAAAAGATCAAAGAGATAACCTCTGAACTGAGCCAGCATGGCCAGATTTCGGA<br>GACTAATGTCTACAATTGGTTCCAGAACAGGCGTGCTCGGTCGAAAAGGAAACAGCAGAATA<br>ATGCAGCACCCAACAATACTGAATCTGAAGTGGAGACTGAAGTTGAGTCACCTAAGGACAAG<br>AAGACAAAGCCGGAGGAGTTTCTGTCCCAACAGAACTCGGCTTCGAGGGCTGAAGATATCTG<br>CTTCCAGAGCTCCGAGATCAGCTCTGACCTGCATTTCTTTGATCCGCATTCCAACAAAGGAGA<br>AGCCATGTTCTCATCAAACAATTCTTTACGGCCTGCGAGGAATTTGACTCAAATGCCATTCTAC<br>GACGGTCTGCTCTCGAACTCAAGAAATGAGCATCTGAGTGGAAAAATGGAAGTGCCAGGGA<br>GTTATGGTCTTTACGAGCATGCGGAAAACTACAGCATGACGGGATGAGGCTATCGTCCGAGGG<br>AGAAGTTAATATTGTGAATAGAAAGAGTACAGATTCCAGATGCAGCTTGGTGGATGTGTTATTA<br>GCAATCATTGCAATTGGGTGCCGTACAAACAGTCTTGGATATGGAAATGTACCTGAACCCACC<br>TACTTAGTAAAGAGTACAAAGCTAACTTAGCAGAGTTTAATGTTTCTCTTTTTGTCTTCATTTTT<br>ATCTGGTCCAGTTTTATCAGGGAGTAGAATGTCATGGTTTTGATTGGTGAACAAACAAACTTGA<br>GTCTCTTTAAGTTCCTGTTTAACTTTTGATAAATGTGAGGACTTTTTGCCCTTTTTGTGTTTTGGC<br>TGATAGTTGATCCGTGAGTTCCTTTTGCTTTTGTCAATACAATGAACCCATTTTATGATTGGATAT<br>GAATGGATGAACC
BpWOX10	AGTGTCAGTACTAAACACTTGCTTGCAATCTAAGGCTGACTCTCTCTCTTTGTTTCTCTTTCTCT<br>GTCTAAGTCAGATTCTCTGACAGAGGTTGAAGATCCAGGGCTTTTTGACAAATGGTTTAACTA<br>ACTCTGGACTGCTCTACTTTTGTTTGTGTCAAGAAACAGGAGTGAGAAAGAGAGCAATTATAG<br>TTAAGGAAGATGATGGAGTGGAAGATTCAACAACAACAACAAGATCATCATGATCATGATCA<br>TGATAAACATGATCATCAGATGGAGATGGGAATATTGCAGAAAAAGGAAGATCAAGAGAATT<br>CAACTATTGTTGGGGTTGTTCCTCCTCCAAATCCAAAAGATGGGTTAGGGTTAATGTACGTGAA<br>GGTTATGACTGACGAGCAAATGGAGCTTCTCAGGCGCCAGATCTCCGTCTACGCCACCATCTG<br>CGAGAAGCTTGCTGACATGTTCAACTCCTTCTCTTCCCAACATGATCTCGCTGGAATGAAGCT<br>GGGAAGTCTTCACTATGATCCACTAATGACATATGGTGGCCACAAGATCACTGCAAGGCAAA<br>GGTGGTCACCAACGCCTGTGCAGCTTCAAACTCTAGAGAAAATTTTCGATGAAGGGAATGGG<br>ACTCCGAGCAAGCAGAAGATCAAAGAGATAACCGTAGAGCTGAGTCAACATGGCCAAATTTC<br>GGAAACCAATGTCTACAATTGGTTCCAGAACAGGAGAGCTCGTTCGAAGAGAAAGCAATCCG<br>TTCCAGCTCCAAACTGCGTGGAACTAGAAGTAGAGACCGAAACTCAAGAGCATTTGAAGGAC<br>AGTGCAAAACCAGAAGATACTGAATTCTGTGACAACTCGGCTGCAGGGGAGGATCATGTGTA<br>TTTTCCAAGTCCTGAGGAGTTGATCAACTGGTCCGCGAATGGAAGTGCCATTGAGCTGCATGC<br>CTTGCCTTCATGGGGAGGATTTGATGTGGATGAGCTCTAGTTGGGATTCTTATATGGTCCTGTTG<br>TTGCTCTTCAGCTGATCTTATTATAAATTTCTGTTCAATGGATTAGAATTTTACATTAGAGAGAA<br>CTTCATTATTAAAATATTATAGTCATAGGTAATTTAGCAATCACTGACAATCTGACATGTGGACT<br>TATAAAATCGTATGTGTCAGACTTGGCTAAAAAACTAAACTGATAGCGAATGTCTTTGATGTTA<br>GCATGTATATGTAATCTTTACAATCAGATTATGATGAACTGGGTCTATTGAAAAACC

Table S5. The species information used to reconstruct the phylogenetic tree. Letter A represents the ancient clade, I represents the intermediate clade, and W represents the WUS clade.

**Species**	**The numbers of WOX gene**	**A**	**I**	**W**
Ostreococcus lucimarinus	1	1	0	0
Physcomitrella patens	3	3	0	0
Selaginella moellendorffii	8	7	1	0
Picea abies	18	4	8	6
Amborella trichopoda	9	1	2	6
Oryza sativa. ssp. Japonica	14	1	6	7
Brachypodium distachyon	13	2	5	6
Populus trichocarpa	18	3	4	11
Medicago truncatula	19	3	7	9
Morus notabilis	11	2	2	7
Broussonetia papyrifera	10	2	2	6
Prunus persica	10	2	2	6
Cucumis melo	10	2	2	6
Vitis vinifera	11	3	2	6
Gossypium raimondii	20	4	4	12
Arabidopsis thaliana	15	3	4	8
Solanum lycopersicum	10	1	2	7
Theobroma cacao	11	2	2	7
Utricularia gibba	17	1	4	12
Total number	228	47	59	59

Table S8. The primer sequences of *BpWOXs* used in qRT-PCR.

Gene Name	Forward primer (5′-3′)	Reverse primer (5′-3′)
BpWUS	TGAGCTAACCCTCAACTCCTACG	TCACACAATCATCATAAATAGAGCATG
BpWOX1	GCCGGCCTACAGATCAGAAAATG	CTTCAATGGTAGGAACTCAAAGAACTG
BpWOX2	ACTGCTACAACTCTTGAGCTATTTCC	ACTCACGTCCTTTATTCTTACTTTGC
BpWOX3	GAAGGAGAACTCCATGATGAGAATG	GAGAGCCATGCAGATAGGAATTTAG
BpWOX4	CTAGTCCGTACAAGAGGAAGTGCC	CATATACCGGCCACATAAACTAAAG
BpWOX5	GCATTAAAGCGGGACATAACATG	GTCCTCTCTTAGAACGAAATCCTTGT
BpWOX7	CGTTAAGTAATAACTGAAGCAGCATCT	ATGGGAGACTGGCATTACAAATAAG
BpWOX8	GACGACGGTGGCAAGGACTAC	AGGCTGGAGGGAGTGAAGAGTG
BpWOX9	ATGCGGAAAACTACAGCATGAC	CAAAACCATGACATTCTACTCCCTG
BpWOX10	CTAGAAGTAGAGACCGAAACTCAAGAGC	CTGAAGAGCAACAACAGGACCAT
BpGAPDH	TCAACATCATTCCTAGCAGTACCG	AGTCAGTGGAAACCACGTCATC

Table S9. The restriction enzymes and primers used to construct the recombinant expression vectors for subcellular localization (SL) and transactivation activity assay (TA).

Gene Name		Forward primer (5′-3′)		Reverse primer (5′-3′)
BpWUS-SL	*Xba*I	GCTCTAGAATGGAACCTCAAC AACAAAACCAATC	*Sal*I	ACGCGTCGACAAGCGAATCCGG TGACCCGTAG
BpWOX1-SL	*Xba*I	GCTCTAGAATGTGGATGATGG GCTATAACGACG	*Sal*I	ACGCGTCGACATTCTTCAATGGT AGGAACTC
BpWOX2-SL	*Xba*I	GCTCTAGAATGTGGACGATGG GTCGTAGTACTA	*Pst*I	AACTGCAGGAGGAAGAAACGCA ACGAGAGG
BpWOX3-SL	*Xba*I	GCTCTAGAATGTCTCCAGCAG CCAATTCATCAAG	*Sal*I	ACGCGTCGACGATGGTCATCGTT GGGGTGGAATG
BpWOX4-SL	*Xba*I	GCTCTAGAATGGGAAGAAGTA GCTGCAGCATGA	*Sal*I	ACGCGTCGACTCTCATGCCTTCC GGGTGTAA
BpWOX5-SL	*Xba*I	GCTCTAGAATGGAAGATCATG GCATGTCAGGCT	*Sal*I	ACGCGTCGACGACAAAGCTTAA GCGCAGAT
BpWOX7-SL	*Xba*I	GCTCTAGAATGGAAGATCATC ACCATCATCAAGG	*Sal*I	ACGCGTCGACGTTATTACTTAAC GTTGTTCT
BpWOX8-SL	*Xba*I	GCTCTAGAATGGCTTCATCAA ACAGACACTGGC	*Sal*I	ACGCGTCGACAAATTAGATAATA AAAAGCACCAGG
BpWOX9-SL	*Xba*I	GCTCTAGAATGATGGGGTGGG AGAAGCAGGAG	*Sal*I	ACGCGTCGACTCCCGTCATGCTG TAGTTTTC
BpWOX10-SL	*Sma*I	TCCCCCGGGATGATGGAGTGG AAGATTCAACAAC	*Sal*I	ACGCGTCGACGAGCTCATCCACA TCAAATCCTC
BpWUS-TA	*Sma*I	TCCCCCGGGGATGGAACCTCA ACAACAAAACCAATC	*Sal*I	ACGCGTCGACGGTTAAAGCGAAT CCGGTGACCCGTAG
BpWOX1-TA	*Sma*I	TCCCCCGGGGATGTGGATGAT GGGCTATAACGACG	*Sal*I	ACGCGTCGACGGTCAATTCTTCA ATGGTAGGAACTC
BpWOX2-TA	*Sma*I	TCCCCCGGGGATGTGGACGAT GGGTCGTAGTACTA	*Pst*I	AACTGCAGCCTCAGAGGAAGAA ACGCAACGAGAGG
BpWOX3-TA	*Sma*I	TCCCCCGGGGATGTCTCCAGC AGCCAATTCATCAAG	*Sal*I	ACGCGTCGACGGTTAGATGGTCA TCGTTGGGGTGGAATG
BpWOX4-TA	*EcoR*I	CCGGAATTCCGGATGGGAAGA AGTAGCTGC	*Sal*I	ACGCGTCGACGGTCATCTCATGC CTTCCGGGTGTAA
BpWOX5-TA	*Sma*I	TCCCCCGGGGATGGAAGATCA TGGCATGTC	*Sal*I	ACGCGTCGACGGTTAGACAAAG CTTAAGCGCAGAT
BpWOX7-TA	*Sma*I	TCCCCCGGGGATGGAAGATCA TCACCATCATCAAGG	*Sal*I	ACGCGTCGACGGTCAGTTATTAC TTAACGTTGTTCT
BpWOX8-TA	*Sma*I	TCCCCCGGGGATGGCTTCATC AAACAGACACTGGC	*Sal*I	ACGCGTCGACGGCTAAATTAGAT AATAAAAAGCACCAGG
BpWOX9-TA	*Sma*I	TCCCCCGGGGATGATGGGGTG GGAGAAGCAGGAG	*Sal*I	ACGCGTCGACGGTCATCCCGTCA TGCTGTAGTTTTC
BpWOX10-TA	*Sma*I	TCCCCCGGGGATGATGGAGTG GAAGATTCAACAAC	*Sal*I	ACGCGTCGACGGCTAGAGCTCAT CCACATCAAATCCTC
